# Development and validation of a real-time computer-aided measuring system for colorectal polyp size (with video)

**DOI:** 10.1093/gastro/goag041

**Published:** 2026-05-12

**Authors:** Cheng-Long Wang, Xiang-Yu Sui, Song Zhang, Jia-Hui Wei, Jia-Yi Wu, Yi-Ta Wu, Xin-Xin Huang, Wen-Long Li, Zi-Ye Zhao, Yi Zeng, Zhi-Wei Liu, Huan-Wei Zhang, You-Dong Zhao, Qi-Wen Fang, Shi-Hang Wang, Xin Li, Kevin Chang, Zhao-Shen Li, En-Da Yu, Sheng-Bing Zhao, Yu Bai

**Affiliations:** Department of Gastroenterology, Changhai Hospital, Naval Medical University, 168 Changhai Road, Shanghai 200433, P. R. China; Department of Colorectal Surgery and Gastrointestinal Endoscopy Center, Changhai Hospital, Naval Medical University, 168 Changhai Road, Shanghai 200433, P. R. China; Department of Gastroenterology, Xiamen University Affiliated Chenggong Hospital (Army 73rd Group Military Hospital), 94 Wenyuan Road, Xiamen, Fujian 361003, P. R. China; Department of Gastroenterology, Changhai Hospital, Naval Medical University, 168 Changhai Road, Shanghai 200433, P. R. China; Department of Gastroenterology, Changhai Hospital, Naval Medical University, 168 Changhai Road, Shanghai 200433, P. R. China; Department of Gastroenterology, Changhai Hospital, Naval Medical University, 168 Changhai Road, Shanghai 200433, P. R. China; Department of Construction Engineering, Jinhua Open University, 18 Qingzhao Road, Jinhua, Zhejiang 321000, P. R. China; AI Solutions Department, Insign Medical Technology (Ningbo) Ltd., 112 Binjiang Road, Cixi, Zhejiang 315300, P. R. China; Department of Gastroenterology, Changhai Hospital, Naval Medical University, 168 Changhai Road, Shanghai 200433, P. R. China; Department of Gastroenterology, Changhai Hospital, Naval Medical University, 168 Changhai Road, Shanghai 200433, P. R. China; Department of Colorectal Surgery and Gastrointestinal Endoscopy Center, Changhai Hospital, Naval Medical University, 168 Changhai Road, Shanghai 200433, P. R. China; Department of Colorectal Surgery and Gastrointestinal Endoscopy Center, Changhai Hospital, Naval Medical University, 168 Changhai Road, Shanghai 200433, P. R. China; Department of Colorectal Surgery and Gastrointestinal Endoscopy Center, Changhai Hospital, Naval Medical University, 168 Changhai Road, Shanghai 200433, P. R. China; Department of Gastroenterology, Changhai Hospital, Naval Medical University, 168 Changhai Road, Shanghai 200433, P. R. China; Department of Gastroenterology, Changhai Hospital, Naval Medical University, 168 Changhai Road, Shanghai 200433, P. R. China; Department of Gastroenterology, Changhai Hospital, Naval Medical University, 168 Changhai Road, Shanghai 200433, P. R. China; Department of Gastroenterology, Changhai Hospital, Naval Medical University, 168 Changhai Road, Shanghai 200433, P. R. China; Department of Gastroenterology, Changhai Hospital, Naval Medical University, 168 Changhai Road, Shanghai 200433, P. R. China; AI Solutions Department, Insign Medical Technology (Ningbo) Ltd., 112 Binjiang Road, Cixi, Zhejiang 315300, P. R. China; Department of Gastroenterology, Changhai Hospital, Naval Medical University, 168 Changhai Road, Shanghai 200433, P. R. China; Department of Colorectal Surgery and Gastrointestinal Endoscopy Center, Changhai Hospital, Naval Medical University, 168 Changhai Road, Shanghai 200433, P. R. China; National Key Laboratory of Immunity and Inflammation, Naval Medical University, 258 Zhongyuan Road, Shanghai 200433, P. R. China; National Clinical Research Center for Digestive Diseases, Naval Medical University, 168 Changhai Road, Shanghai 200433, P. R. China

**Keywords:** artificial intelligence, polyp size, deep learning, colonoscopy, depth map

## Abstract

**Background:**

Polyp size measurement is critical in determining the resection method and surveillance interval; however, the accuracy and reliability of existing measurement methods are suboptimal. We establish a computer-aided measuring (CAM) system to automatically and accurately measure colorectal polyp size in real time.

**Methods:**

We conducted a hybrid retrospective and prospective preclinical study to develop models retrospectively and validate them prospectively. The CAM system includes two deep learning models: a polyp detection model trained on 15,620 images and tested on 4,438 images and a depth-map prediction model trained on 18,500 red–green–blue depth-map pairs and tested on 1,500 pairs. A digitized three-dimensional model development setting (3D-MDS) was used to validate the CAM system; here, 3,750 red–green–blue images from 15 digital virtual 3D polyps were generated for size measurements. A high-simulation colon/polyp model was used to test real-time polyp size measurement performance.

**Results:**

In the 3D-MDS, the CAM system had an average relative error of 7.9% (standard deviation 4.19%) for the depth-map prediction model and a mean percentage error of 7.9% (interquartile range [IQR] 4.6%–10.4%) for the polyp size measurement. In the high-simulation colon/polyp model, the CAM system’s depth distance measurement had a percentage error of 12.0% (IQR 5.5%–15.7%). For the polyp size measurement, the highest accuracy and reliability were achieved at the medium-depth distance and the position within the center field-of-view, with percentage errors of 4.1% (IQR 2.6%–4.5%) and 3.3% (IQR 1.8%–3.5%) for the first and second measurements, respectively, and an intraclass correlation coefficient of 0.999.

**Conclusions:**

The CAM system showed excellent accuracy and reliability in diverse testing scenarios; here, the medium-depth distance (15–35 mm) and the position within the center field-of-view was likely the optimal measurement location.

## Introduction

Colorectal cancer (CRC) ranks third worldwide among cancers and second in terms of cancer-related deaths [1, 2]. It also ranks third in terms of the global burden of digestive diseases [3]. Polyp size is an important predictor of colorectal cancer risk and surveillance frequency and is closely related to the choice of resection type and patient prognosis [4, 5]. Therefore, accurate measurement of polyp size is crucial for clinical decision-making [6, 7]. It directly dictates the choice of resection technique (e.g. cold snare polypectomy for sub-1 cm polyps vs endoscopic mucosal resection for larger lesions) and determines post-polypectomy surveillance intervals according to major guidelines (e.g. the 2020 European Society of Gastrointestinal Endoscopy guideline [5], which stratifies risk based on polyp size ≥ 10 mm). Inaccurate sizing can thus lead to inappropriate resection methods, increasing the risk of adverse events like perforation or incomplete resection, and result in misguided surveillance schedules, potentially missing early cancer development or causing unnecessary frequent colonoscopies [4].

Despite advancements in endoscopic measurement tools, including biopsy forceps [8–11], transparent caps [12–14], graduated sheaths [15, 16], laser probes [17], and laser-based virtual scale endoscopes [18–20], these instruments still fall short in terms of overall accuracy, increased costs, and the need for extra measurement-related procedures, to a certain extent. Regardless of the auxiliary tools, the current practice of measuring polyp size relies largely on the subjective assessment by the endoscopist, and this assessment suffers from low accuracy and inter- or intra-observer agreement. Moreover, the endoscopic “fish-eye” effect and variability of the colon may further aggravate the difficulty in measuring polyp size [21–25]. Measurement of fresh or formalin-fixed polyps immediately after removal was once considered a rational alternative; however, its accuracy is also influenced by alterations in polyp shape and size due to piecemeal resection or suction through endoscopic channels [21, 22]. Several of the latest systems that were integrated with artificial intelligence (AI) have provided further advancements and were reported to show relatively high accuracy and agreement [26–30]. Nevertheless, owing to the dependence on reference media, including branches of the main blood vessels [26], auxiliary water jets [27], and biopsy forceps [28, 29], or nonreal-time measurements [26, 30], these methods are not purely intelligent and generalizable systems for measuring polyp size.

In this study, we develop a computer-aided measuring (CAM) system (ENDODASS) for real-time automated polyp size measurement without extra auxiliary instruments or reference devices. A digitized three-dimensional model development setting (3D-MDS) and high-simulation colon/polyp model were used to train and validate the CAM system.

## Methods

This study is reported according to the quality assessment of AI preclinical studies in diagnostic endoscopy (QUAIDE) statement [31]. Institutional approval is not required as no human participants or animals are involved.

### Study overview

**Figure 1:**
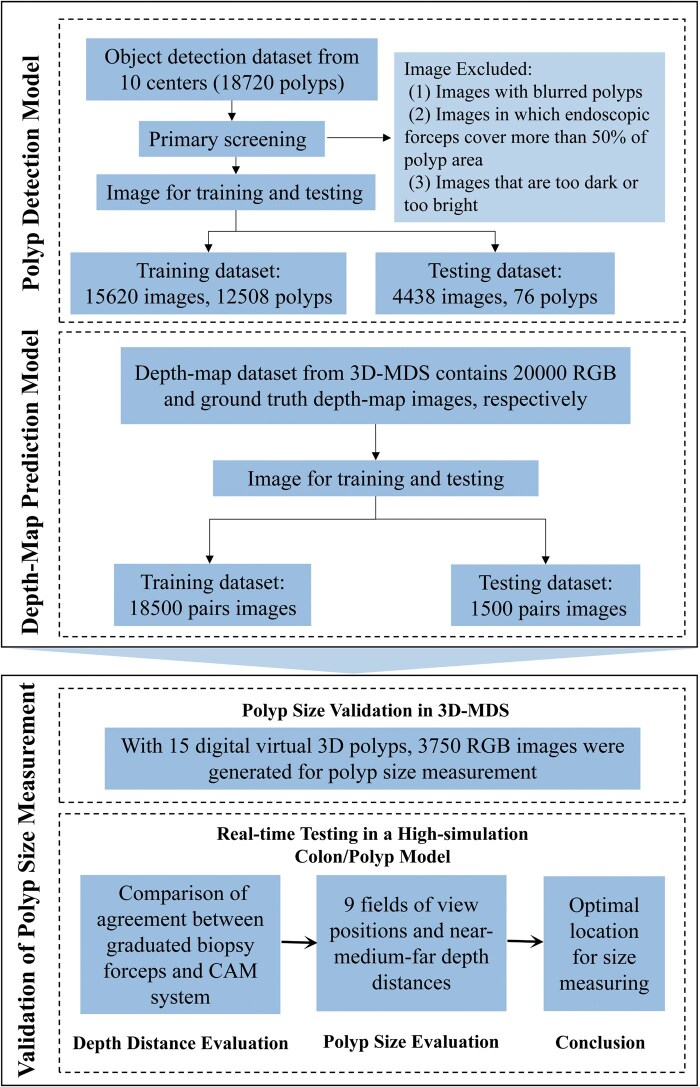
Total flowchart of study. 3D, three-dimensional; 3D-MDS, digitized 3D model development setting; CAM, computer-aided measuring; RGB, red-green-blue.

The study design is a combined retrospective (for model development) and prospective (for system validation) preclinical study. Two primary deep learning-based models were employed and integrated into the construction of a CAM system: a polyp detection model and a depth-map prediction model for polyp size measurement. Once a polyp is detected by the polyp detection model, the CAM system initiates the depth-map prediction model to predict the depth distance and calculate the initial polyp size. The initial measurement is then adjusted via colonoscopy lens parameters and a fine-tuning procedure to increase the polyp size estimation accuracy. During the process, the CAM system uses 3D computed tomography (CT) reconstruction to (i) generate a ground truth depth map for training the depth-map prediction model for predicting the depth distance from the endoscopic lens to the polyp surface and (ii) validate the CAM system. The performance of the CAM system was tested with a high-simulation colon/polyp model. A comprehensive study flowchart is shown in Fig. 1, with further details available in Supplementary Video 1.

### Polyp detection model

**Figure 2:**
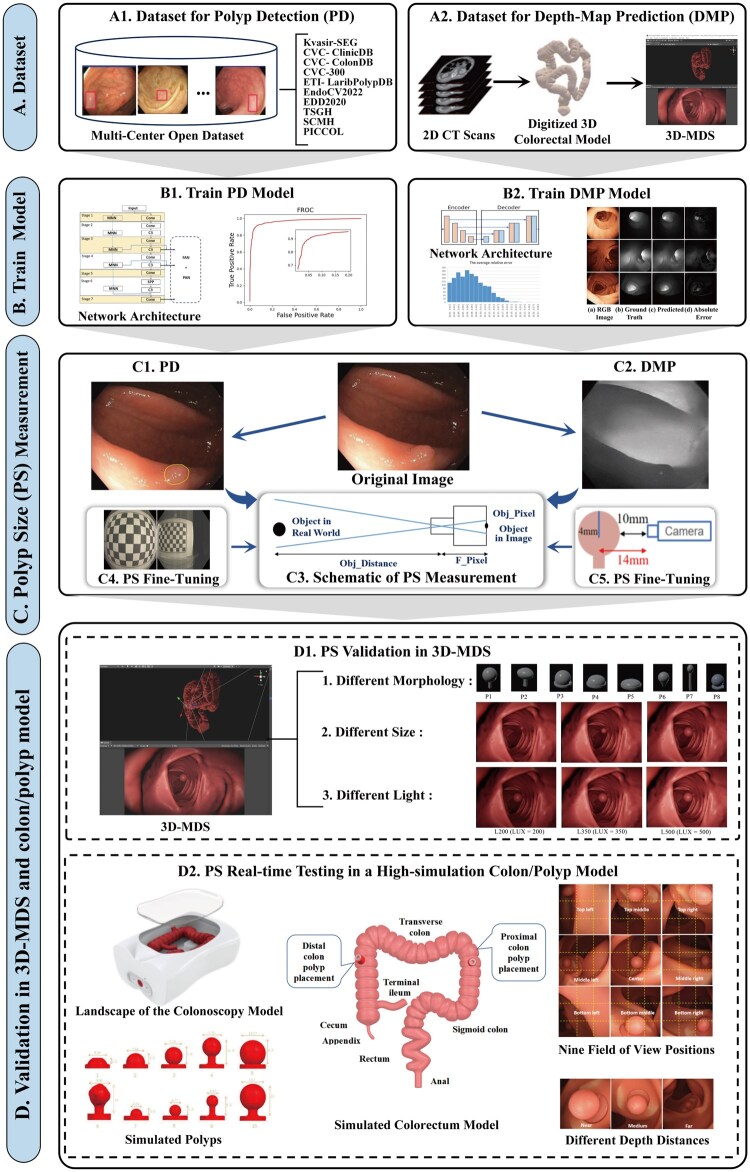
Total framework of polyp size measurement. (A) Dataset collection including open source for PD (A1) and digitized 3D-MDS for DMP (A2). (B) Model training containing PD model (B1) and DMP model (B2). (C) PS measurement containing PD (C1), DMP (C2), Schematic of PS measurement (C3), PS fine-turning procedure (C4 and C5). (D) Validation containing PS validation in 3D-MDS (D1) and PS real-time testing in high-simulation colon/polyp model (D2). Details of the 10 dataset materials for PD are provided in supplementary materials. 2D, two-dimensional; 3D, three-dimensional; 3D-MDS, 3D model development setting; CT, computed tomography; DMP, depth-map prediction; FROC, free-receiver operating characteristic; PD, polyp detection; PS, polyp size; RGB, red-green-blue.

In our previous study [32], a polyp detection model was developed based on a deep hybrid neural network to detect the position and region of interest of polyps in the image frame (Fig. 2B1 and C1 and Supplementary Fig. 1), significantly impacting the identification, localization, and diagnostic accuracies of polyps in the CAM system. This model was trained and tested with 15,620 and 4,438 polyp images, respectively (Fig. 1). More details are provided in the Supplementary Material.

### Depth-map prediction model

#### Model construction

The depth-map prediction model employs an encoder–decoder architecture neural network [33] to predict depth maps corresponding to the same image frame (Fig. 2B2 and C2), which is crucial for predicting the depth distance of a polyp as the polyp detection model detects the polyp. The synergy between this model and the polyp detection model is critical for accurate and efficient polyp size measurement. The depth-map prediction model was trained on 18,500 pairs of red–green–blue (RGB) and ground truth depth-map images and subsequently tested on 1,500 image pairs using 3D-CT reconstruction techniques (Fig. 1). Additional details are provided in the Supplementary Material.

#### 3D-CT reconstruction technique for training

To collect a ground truth dataset for training the depth-map prediction model, we used the 3D-CT reconstruction technique to convert 2D images into 3D images, thus establishing a digitized virtual 3D colorectal model. Specifically, we first processed 2D CT image data for visualization, selecting the region of interest in the colorectum and adjusting its surface texture and internal pathways. Next, we created a digitized virtual 3D colorectal model and imported it into Unity (v3.5.0, Unity Technologies, USA), a real-time 3D authoring platform, creating a workspace named 3D-MDS (Fig. 2A2). Within the 3D-MDS, by using Unity’s 3D editing tools and robust scene editor to set relevant parameters, such as the camera field of view (FOV) and virtual light source illumination, we completed the collection of the ground truth dataset required for depth-map prediction model training.

### Polyp size measurement and fine-tuning procedure

#### Initial measurement

After the polyp position and corresponding depth map obtained through the above two deep learning models are integrated, the initial estimated polyp size can be determined. The principle of “triangle similarity” in determining polyp size is illustrated in Fig. 2C3. Assuming that the size of an object detected in the image (in pixels) is *Obj_Pixel*, the distance of the object is *Obj_Distance*, and the camera focal length is *F_Pixel*, the initial estimated size of the object can be calculated using Equation 1:


(1) $$\text{Object}\_\text{Size}=\frac{\text{Obj}\_\text{Distance}*\text{Obj}\_\text{Pixel}}{F\_\text{Pixel}}$$

**Figure 3:**
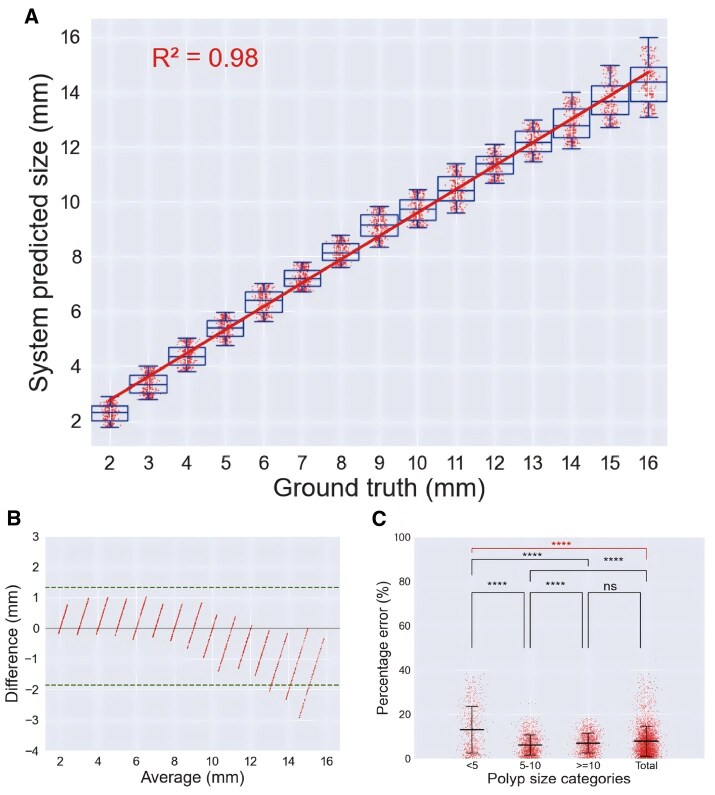
Validation of polyp size measurement in the digitized three-dimensional model development setting. (A) Box and scatter plot with linear regression trend of system. (B) Bland–Altman plots of the system. (C) Percentage error in polyp size categories according to the cut-off of 5 and 10 mm. ns, not significant; ****P < 0.0001.

#### “Fish-eye” effect

In this study, we used an Olympus 170° fish-eye lens. This lens exhibits significant lens distortion, causing noticeable differences in the size and position of objects in the image (Fig. 2C4). Assuming that *FOV_Fish-eye* is the FOV of the fish-eye camera and that *FOV_Normal* is the FOV of the normal camera, the corrected size of an object detected in the image can be obtained as follows:


(2) $$\text{Obj}\_\text{Pixel}\_\text{New}=\frac{\text{arctan}(\frac{\text{Ob}j_{\text{Pixel}}}{\text{Ob}j_{\text{Distance}}})*\text{FOV}\_\text{Fish}-\text{eye}}{\text{FOV}\_\text{Normal}}$$

**Figure 4:**
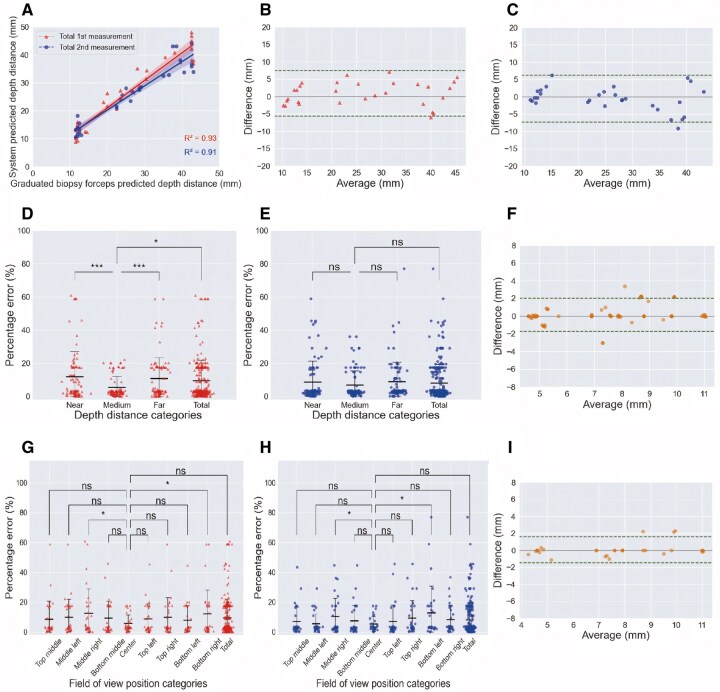
Real-time performance of CAM system in the high-simulation colon/polyp model. (A)–(C) Real-time performance of depth distance measurement: (A) Scatter plot with linear regression trend of the system showing comparison of the performance between total 1st and 2nd measurements; (B) and (C) The performance of total 1st measurement and 2nd measurement using Bland–Altman plots of the system, respectively. (D)–(I) Real-time performance of polyp size measurement: (D), (E), (G) and (H) Percentage errors of the 1st and 2nd measurements according to different field of view positions or depth distances, respectively; (F) and (I) Bland–Altman plots of the system showing comparison of the performance between the 1st and 2nd measurements by medium-depth distance or center-field of view position, respectively. ns, not significant; *P < 0.05; ***P < 0.001. CAM, computer-aided measuring.

#### “Polyp-thickness” effect

The depth-map prediction model estimates the distance between the polyp surface and the endoscopic lens; thus, the polyp thickness will cause a certain amount of error. In general, this type of error caused by object thickness is usually disregarded. In endoscopic images, both the depth distance and polyp size are measured in millimeters; thus, this error in the polyp size estimation should not be disregarded and is adjusted in our measurement (Equation 3). For instance, the polyp size and estimated depth distance were 8 and 10 mm, respectively. After considering a polyp radius of 4 mm, the true depth distance should be 14 mm, resulting in a 29% error (Fig. 2C5).

**Figure 5:**
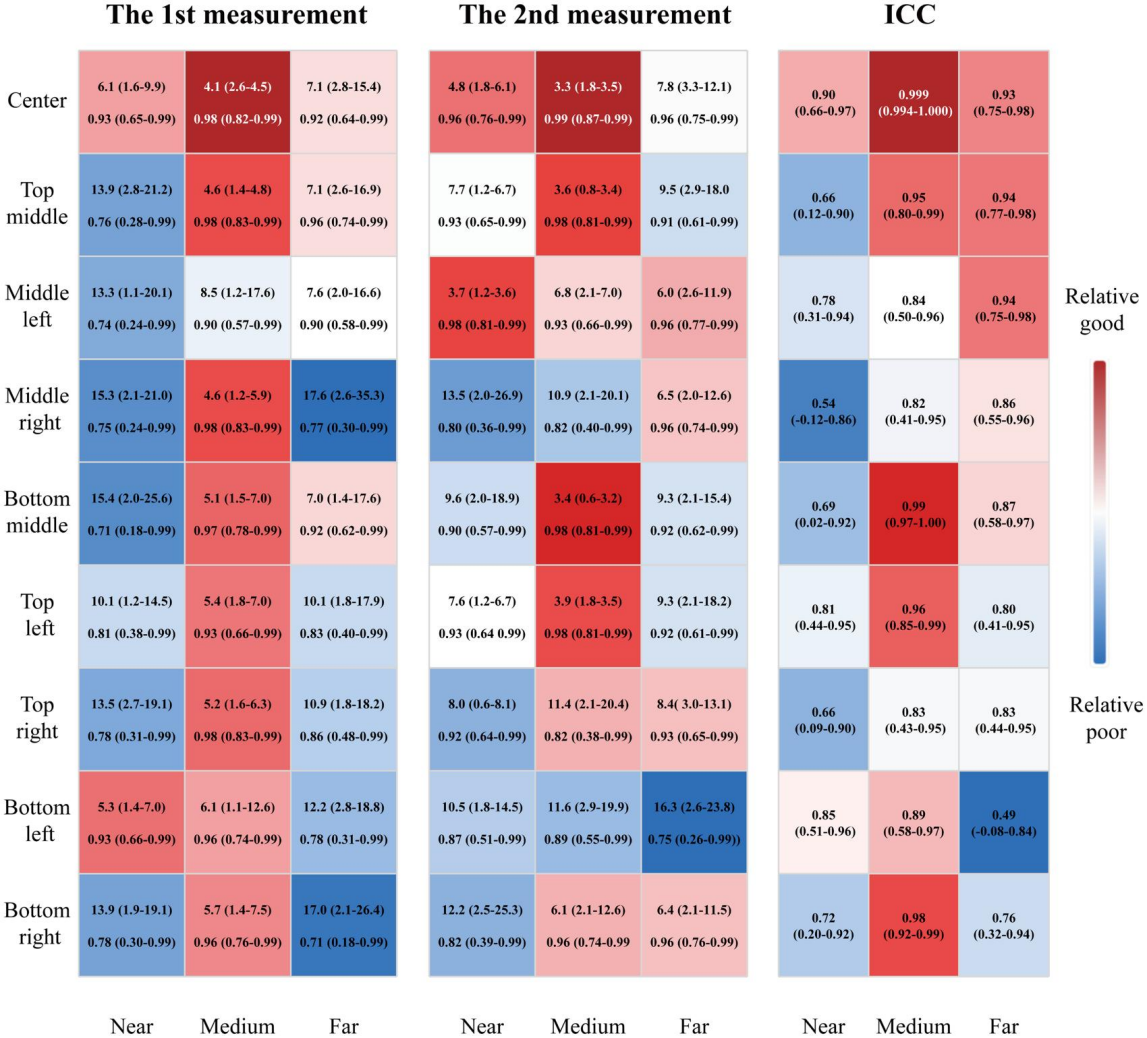
Real-time performance of polyp size measurement in the high-simulation colon/polyp model by near, medium, and far depth distances and 9 fields of view positions. The 1st measurement: the percentage error (mean [interquartile range], %) and Lin’s concordance correlation coefficient (95% confidence interval) of the 1st measurement of polyp size; The 2nd measurement: the percentage error (mean [interquartile range], %) and Lin’s concordance correlation coefficient (95% confidence interval) of the 2nd measurement of polyp size. ICC, the intraclass correlation coefficient (95% confidence interval) between the polyp size measurement of the 1st and 2nd measurements.

#### Final measurement

The final polyp size is determined after fine-tuning procedures for the “fish-eye” and “polyp-thickness” effects. Therefore, the depth distance is subsequently recalculated via the fine-tuning procedure using the polyp radius (*Obj_Radius*) and the corrected size of the object detected in the image (*Obj_Pixel_New)* to obtain the final predicted polyp size via the triangle similarity method as follows:


(3) $${O\text{bject}\_\text{Size}}_{F\text{inal}}=\frac{(\text{Obj}\_\text{Distance}+\text{Obj}\_\text{Radius})*\text{Obj}\_\text{Pixel}\_\text{New}}{F\_\text{Pixel}}$$

### Validation of the CAM system

#### Validation of the polyp size measurement via 3D-MDS

Through a digitized virtual 3D colorectal model and 3D-MDS, we generated 15 pedunculated or flat digital virtual 3D polyps with maximum diameters ranging from 2 to 16 mm. For each polyp, we created 250 images with different brightness and colorectal positions for measurement validation. An intuitive representation is shown in Fig. 2D1 and Supplementary Fig. 2.

##### Real-time testing in a high-simulation colon/polyp model

###### Material preparation

Ten simulated polyps with diameter sizes ranging from 4 to 11 mm were created, four with polyp morphology and regular and irregular shapes. The ground-truth diameter of the polyp was measured using vernier calipers. An intuitive representation is shown in Fig. 2D2 and Supplementary Figs. 3–5, and an overview of the model and simulated polyps can be found in Supplementary Video 2.

A transparent film with nine FOVs was placed on the screen, and the specific viewing position of the simulated polyp was determined by two factors: depth distance (distance from the lens to the center of the polyp) and FOV position; there were 9 FOV positions and near (< 15 mm), medium (15–35 mm), and far (> 35 mm) depth distances. Notably, based on our systematic pre-testing results and the commonly accepted depth distances by most endoscopists, we statistically determined that a suitable medium-depth distance ranges from 15 to 35 mm. Consequently, we classified depth distance into three categories: near, medium, and far. A visualization is shown in Fig. 2D2.

### Procedures

Supplementary Figure 6 shows that endoscopic depth distance measurements taken by graduated biopsy forceps and the CAM system via autonomous AI measurement [34] (Supplementary Material) were performed on 10 simulated polyps at the center-FOV position and the near, medium, and far depth distances twice, 1 week apart, and the data were checked and validated by three mutually independent researchers.

Autonomous AI measurements of 10 simulated polyps determined by near-, medium-, and far-depth distances and 9 FOV positions were also performed twice, 1 week apart, and the data were checked and validated by three mutually independent researchers. An intuitive representation is shown in Supplementary Fig. 7.

### Outcomes

The primary outcome was the percentage error (PE) in predicting polyp size. The secondary outcomes were the PE in predicting the depth distance; Lin’s concordance correlation coefficient (CCC) between the polyp size measurement of the system and the ground truth; the CCC between the depth distance measurement of the system and the graduated biopsy forceps; the intraclass correlation coefficient (ICC) between the first and second polyp size measurements; the measurement error (ME) between the polyp size measurement of the system and the ground truth; and the ME between the depth distance measurement of the system and the graduated biopsy forceps.

### Statistical analysis

The average relative error (ARE) of the CAM system in estimating the depth distance in 3D-MDS is shown in Equation 4, where $d_{\text{GT}}$ and $d_{\text{CAM}}$ are the ground truth and system-predicted depth-map image, respectively, and *n* is the number of pixels of each depth-map image.


(4) $$A\text{RE}=\frac{1}{n}\sum_{1}^{n}\frac{|d_{\text{GT}}-d_{\text{CAM}}|}{d_{GT}}$$

The root mean square error (RMSE) of the system in estimating the depth distance in 3D-MDS is shown in Equation 5.


(5) $$\text{RMSE}=\sqrt{\frac{1}{n}\sum_{1}^{n}{\left(d_{\text{GT}}-d_{\text{CAM}}\right)}^{2}}$$

The average ($\;{\text{log}}_{10}$) error of the system in estimating the depth distance in 3D-MDS is shown in Equation 6.


(6) $${\text{AveE}\_\;\text{log}\;}_{10}=\frac{1}{n}\sum_{1}^{n}|\;{\text{log}}_{10}(d_{\text{GT}})-{\text{log}}_{10}(d_{\text{CAM}})|$$

The percentage error of the system in estimating the polyp size (${\text{PE}}_{\text{Size}}$) is shown in Equation 7, where ${\text{PSize}}_{\text{CAM}}$ and ${\text{PSize}}_{\text{GT}}$ are the autonomous AI measurement and ground truth polyp sizes, respectively. Note that the difference between ${\text{PSize}}_{\text{CAM}}$ and ${\text{PSize}}_{\text{GT}}$ is defined as the measurement error (${\text{ME}}_{\text{Size}}$) of the system, as shown in Equation 8.


(7) $${\text{PE}}_{\text{Size}}=\frac{|{\text{PSize}}_{\text{CAM}}-{\text{PSize}}_{\text{GT}}|}{{\text{PSize}}_{\text{GT}}}*100\%$$

(8) $${\text{ME}}_{\text{Size}}={\text{PSize}}_{\text{CAM}}-{\text{PSize}}_{\text{GT}}$$

The percentage error (${\text{PE}}_{\text{Depth}}$) of the system in estimating the depth distance in the high-simulation colonoscopy model is shown in Equation 9, where ${\text{PDepth}}_{\text{CAM}}$ and ${\text{PDepth}}_{\text{GBF}}$ are the depth distances predicted by the system and graduated biopsy forceps, respectively. Note that the difference between ${\text{PDepth}}_{\text{CAM}}$ and ${\text{PDepth}}_{\text{GBF}}$ is defined as the measurement error (${\text{ME}}_{\text{Depth}}$) of the system, as shown in Equation 10.


(9) $${\text{PE}}_{\text{Depth}}=\frac{|{\text{PDepth}}_{\text{CAM}}-{\text{PDepth}}_{\text{GBF}}|}{{\text{PDepth}}_{\text{GBF}}}*100\%$$

(10) $${\text{ME}}_{\text{Depth}}={P\text{Depth}}_{\text{CAM}}-{P\text{Depth}}_{G\text{BF}}$$

This work was conducted to develop and preliminarily validate a new algorithm. Since the objective was not therapeutic hypothesis testing, conventional sample size estimation methods were not applicable. The performance of the CAM system was evaluated via several metrics, including the PE, CCC, ICC, and ME. We calculated the CCC and graphically displayed the agreements in Bland–Altman plots [35–37]. To assess the intra-observer agreement between the polyp size measurements, we evaluated the test–retest reliability and calculated the ICC using a two-way mixed effects model and absolute agreement [38, 39]. Additionally, a linear regression trend analysis was conducted to explore whether the system could predict the polyp size or depth distance; an *R*^2^ close to 1 implied an almost perfect relationship between the system and ground truth. The difference in the mean PE of polyp size or depth distance measurement between groups was compared using paired Wilcoxon tests or Mann–Whitney tests, with *P *< 0.05 indicating that there was a significant difference. All data processing and analyses were performed using SPSS 25 (IBM, Chicago, Illinois, USA) and Python 3.12.3 (Python Software Foundation, Wilmington, USA).

## Results

### Performance of the depth-map prediction model

A total of 1,500 pairs of RGB and ground truth depth-map images were generated for evaluation. The experimental results revealed that the ARE, RMSE, and average ($\;{\text{log}}_{10}$) error of the system were 7.9% (standard deviation 4.19%), 4.12 (standard deviation 2.78) mm, and 0.035 (standard deviation 0.021) mm, respectively. Figure 2B2 and Supplementary Fig. 8 show the depth-map prediction results, including the RGB images, ground truth depth map, predicted depth map, and absolute errors of the ground truth and predicted depth maps, respectively.

### Validation in 3D-MDS

The polyp size measurement of the CAM system on 3,750 RGB images of 15 digital virtual 3D polyps generated using 3D-MDS had an *R*^2^ of 0.98 for the ground truth, and the Bland–Altman plot indicated that most of the sample points were more centrally distributed, with limits of agreement ranging from -1.9 to 1.3 mm. The mean PE_Size_ for 15 digital virtual 3D polyps was 7.9% [interquartile range (IQR) 4.6%–10.4%], with a higher PE_Size_ for small polyps (< 5 mm) [small vs total, 13.1% (IQR 3.8%–21.5%) vs 7.9% (IQR 3.0%–10.8%); *P *< 0.0001].

Overall, the CAM system misclassified 271 out of 3,750 RGB images (7.2%); 39 out of 3,012 (1.3%) RGB images with sizes ≥ 5 mm were underestimated as < 5 mm, and 5 out of 738 (0.7%) RGB images < 5 mm were overestimated as ≥ 5 mm. Additionally, 227 out of 1,757 RGB images (12.9%) with sizes ≥ 10 mm were underestimated as < 10 mm, whereas none of the 1,993 RGB images with sizes < 10 mm were overestimated as ≥ 10 mm. Furthermore, the system predicted 15 out of 3,750 RGB images (0.4%) as the terminal digit preference of 5 mm, 10 mm, and 15 mm. In 3D-MDS, the system exhibited stable performance across various brightness, colorectal positions, sizes and morphologies of polyps. An intuitive representation is shown in Fig. 3 and Supplementary Tables 1 and 2.

### Baseline characteristics of simulated polyps and real-time performance of the CAM system for depth distance measurement

Supplementary Table 3 presents detailed information on the maximum diameter, size category, morphology, location, shape, and height of the 10 simulated polyps. The mean size and height of the polyps were 7.5 (IQR 4.9–9.5) mm and 7.7 (IQR 4.1–11.5) mm, respectively. A baseline analysis of the simulated polyps is shown in Supplementary Table 4.

In the analyses of the total first and second measurements, when the measurement results of the graduated biopsy forceps were used as references, the depth distance measurements of the CAM system had ${\text{PE}}_{\text{Depth}}$ values of 13.2% (IQR 7.2%–17.8%) and 10.7% (IQR 3.5%–14.7%), respectively, and CCC values of 0.96 (95% CI 0.85–0.99) and 0.95 (95% CI 0.83–0.99), respectively. In addition, when the total first and second measurements were combined, the ${\text{PE}}_{\text{Depth}}$ was 12.0% (IQR 5.5%–15.7%) and the CCC was 0.96 (95% CI 0.88–0.99). In the subgroup analysis combining the first and second measurements, the medium-depth distance measurement had the lowest ${\text{PE}}_{\text{Depth}}$ of 9.1% (IQR 2.7%–12.6%) and the highest CCC of 0.71 (95% CI 0.38–0.99) (Fig. 4A–C and Supplementary Tables 5 and 6).

### Real-time performance of the CAM system for polyp size measurement

#### Different depth distances

Table 1 provides the results of the subgroup analysis at near-, medium-, and far-depth distances, and a visual representation is shown in Fig. 4D–F; here, the CAM system exhibited good ${\text{PE}}_{\text{Size}}$, CCC, and ICC across all subgroups. Notably, at the medium-depth distance, the CAM system had the lowest$\;{\text{PE}}_{\text{Size}}$for the first and second measurements, at 5.5% (IQR 1.4%–3.7%) and 6.8% (IQR 1.9%–7.6%), respectively. The CCC at the medium-depth distance was the highest in the first measurement and tied for the highest in the second measurement, at 0.96 (95% CI 0.90–0.99) and 0.92 (95% CI 0.84–0.99), respectively. The ICC at the medium-depth distance was also tied for the highest at 0.92 (95% CI 0.84–0.99). Supplementary Table 7 shows that the medium-depth distance yielded the lowest misclassification rates with 8 (14.8%) polyps in the first measurement and 4 (16.7%) polyps in the second measurement, respectively. Supplementary Table 8 provides similar results in the subgroup analysis of the average results.

**Table 1:** Results of real-time polyp size measurement by near, medium, and far depth distances.

Depth distance	Ground truth, mm, mean (IQR)	The 1st measurement					The 2nd measurement					ICC (95% CI)
		CAM system measurement, mm, mean (IQR)	${\text{ME}}_{\text{Size}}$ , mm, mean (IQR)	${\text{PE}}_{\text{Size}}$ , %, mean (IQR)	*P* value	CCC (95% CI)	CAM system measurement, mm, mean (IQR)	${\text{ME}}_{\text{Size}}$ , mm, mean (IQR)	${\text{PE}}_{\text{Size}}$ , %, mean (IQR)	*P* value	CCC (95% CI)	
Near	7.6 (5.0–9.1)	7.5 (5.7–8.8)	0.9 (0.2–1.5)	11.9 (2.1–17.8)	< 0.001	0.80 (0.67–0.92)	7.8 (5.0–9.8)	0.6 (0.1–1.1)	8.6 (1.8–13.5)	0.58	0.90 (0.81–0.99)	0.71 (0.59–0.80)
Medium		7.7 (5.7–9.0)	0.4 (0.1–0.3)	5.5 (1.4–3.7)	Reference	0.96 (0.90–0.99)	7.8 (5.5–9.8)	0.5 (0.1–0.5)	6.8 (1.9–7.6)	Reference	0.92 (0.84–0.99)	0.91 (0.87–0.94)
Far		7.6 (6.5–8.8)	0.7 (0.2–1.5)	10.7 (2.1–17.6)	< 0.001	0.86 (0.75–0.97)	7.8 (5.7–9.8)	0.6 (0.2–0.9)	8.8 (2.8–11.9)	0.07	0.92 (0.84–0.99)	0.83 (0.75-0.89)
Total	7.6 (5.0–9.1)	7.6 (5.7–8.8)	0.7 (0.2–1.0)	9.4 (1.9–17.5)	0.016	0.87 (0.82–0.93)	7.8 (5.7–9.8)	0.6 (0.2–0.9)	8.3 (2.1–13.5)	0.25	0.91 (0.86–0.96)	0.82 (0.77–0.85)

IQR, interquartile range; CAM, computer-aided measuring; ${\text{ME}}_{\text{Size}}$, measurement error of polyp size measurement; ${\text{PE}}_{\text{Size}}$, percentage error of polyp size measurement; CCC, Lin’s concordance correlation coefficient between the polyp size measurement of CAM system and ground truth; ICC, intraclass correlation coefficient between the polyp size measurement of the 1st and 2nd measurements; CI, confidence interval.

#### Different FOV positions

Table 2 lists the results of the subgroup analysis at 9 FOV positions and an intuitive representation is shown in Fig. 4G–I; here, the CAM system demonstrated good results for ${\text{PE}}_{\text{Size}}$, CCC, and ICC across all subgroups. The CAM system had the lowest ${\text{PE}}_{\text{Size}}$ at the central-FOV position in both the first and second measurements, with 5.8% (IQR 2.1%–7.8%) and 5.3% (IQR 2.1%–7.9%), respectively. The CCC at the central-FOV position was the highest in the two measurements, at 0.95 (95% CI 0.82–0.99) and 0.97 (95% CI 0.88–0.99), respectively. The ICC at the central-FOV position was also the highest, at 0.94 (95% CI 0.88–0.97). Supplementary Table 7 shows that the lowest misclassification rates, with 2 (3.7%) polyps in the first measurement and zero polyps in the second measurement, were achieved with the center-FOV position. Supplementary Table 9 shows similar results in the subgroup analysis of the average measurements.

**Table 2:** Results of real-time polyp size measurement by 9 field of view positions.

Field of view position	Ground truth, mm, mean (IQR)	The 1st measurement					The 2nd measurement					ICC (95% CI)
		CAM system measurement, mm, mean (IQR)	${\text{ME}}_{\text{Size}}$ , mm, mean (IQR)	${\text{PE}}_{\text{Size}}$ , %, mean (IQR)	*P* value	CCC (95% CI)	CAM system measurement, mm, mean (IQR)	${\text{ME}}_{\text{Size}}$ , mm, mean (IQR)	${\text{PE}}_{\text{Size}}$ , %, mean (IQR)	*P* value	CCC (95% CI)	
Center	7.6 (5.0–9.1)	7.5 (4.8–8.8)	0.5 (0.2–0.5)	5.8 (2.1–7.8)	Reference	0.95 (0.82–0.99)	7.6 (4.8–9.8)	0.4 (0.1–0.5)	5.3 (2.1–7.9)	Reference	0.97 (0.88–0.99)	0.94 (0.88–0.97)
Top middle		7.7 (5.7–8.8)	0.6 (0.2–0.8)	8.5 (2.1–11.0)	0.23	0.90 (0.74–0.99)	7.9 (5.5–9.8)	0.5 (0.1–0.4)	6.9 (2.1–5.4)	0.35	0.94 (0.81–0.99)	0.85 (0.72–0.93)
Middle left		7.5 (5.8–8.8)	0.7 (0.1–1.5)	9.8 (1.4–17.5)	0.07	0.85 (0.65–0.99)	7.8 (4.9–9.8)	0.4 (0.1–0.3)	5.5 (2.1–3.6)	0.88	0.96 (0.85–0.99)	0.84 (0.70–0.92)
Middle right		7.8 (6.5–8.8)	0.8 (0.2–1.3)	12.5 (2.1–18.4)	0.03	0.84 (0.63–0.99)	7.6 (5.5–9.8)	0.8 (0.1–1.5)	10.3 (2.1–17.5)	0.04	0.86 (0.67–0.99)	0.74 (0.52–0.87)
Bottom middle		7.4 (4.9–9.1)	0.7 (0.1–1.1)	9.2 (1.5–17.6)	0.08	0.85 (0.65–0.99)	8.0 (5.5–9.8)	0.5 (0.1–0.8)	7.5 (1.8–13.8)	0.28	0.93 (0.79–0.99)	0.82 (0.62–0.92)
Top left		7.4 (5.5–8.8)	0.7 (0.1–1.5)	8.8 (1.8–17.5)	0.11	0.86 (0.67–0.99)	7.8 (5.5–9.8)	0.5 (0.1–0.3)	7.0 (1.9–3.6)	0.42	0.94 (0.81–0.99)	0.86 (0.71–0.93)
Top right		7.7 (6.5–8.8)	0.7 (0.2–1.0)	9.9 (2.1–17.6)	0.09	0.88 (0.70– 0.99)	7.9 (5.7–9.8)	0.7 (0.2–0.9)	9.3 (2.1–15.4)	0.10	0.89 (0.71-0.99)	0.76 (0.56–0.88)
Bottom left		7.5 (5.7–8.8)	0.6 (0.1–1.0)	7.8 (1.4–17.5)	0.26	0.90 (0.74–0.99)	8.2 (6.9–10.1)	0.8 (0.2–1.5)	12.8 (2.8–17.5)	0.04	0.84 (0.63–0.99)	0.77 (0.51–0.89)
Bottom right		7.9 (6.9–9.1)	0.8 (0.2–1.5)	12.2 (2.1–18.4)	0.03	0.83 (0.62–0.99)	7.6 (5.0–9.8)	0.6 (0.1–1.0)	8.2 (2.1–15.1)	0.11	0.91 (0.75–0.99)	0.82 (0.65–0.91)
Total	7.6 (5.0–9.1)	7.6 (5.7–8.8)	0.7 (0.2–1.0)	9.4 (1.9–17.5)	0.59	0.87 (0.82–0.93)	7.8 (5.7–9.8)	0.6 (0.2–0.9)	8.3 (2.1–13.5)	0.68	0.91 (0.86–0.96)	0.82 (0.77–0.85)

IQR, interquartile range; CAM, computer-aided measuring; ${\text{ME}}_{\text{Size}}$, measurement error of polyp size measurement; ${\text{PE}}_{\text{Size}}$, percentage error of polyp size measurement; CCC, Lin’s concordance correlation coefficient between the polyp size measurement of CAM system and ground truth; ICC, intraclass correlation coefficient between the polyp size measurement of the 1st and 2nd measurements; CI, confidence interval.

#### Different depth distances and within the FOV positions

Figure 5 and Supplementary Table 8 show the analysis results determined by the near-, medium-, and far-depth distances and 9 FOV positions. The CAM system achieved satisfactory ${\text{PE}}_{\text{Size}}$, CCC, and ICC results across all measurement locations. Notably, at the medium-depth distance and within the center-FOV position, the CAM system presented the lowest${\;\text{PE}}_{\text{Size}}$in both the first and second measurements, with values of 4.1% (IQR 2.6%–4.5%) and 3.3% (IQR 1.8%–3.5%), respectively. The CCCs at the medium-depth distance and within the center-FOV position was the highest in the two measurements, with values of 0.98 (95% CI 0.82–0.99) and 0.99 (95% CI 0.87–0.99), respectively. The ICCs at the medium-depth distance and within the center-FOV position was also the highest, at 0.999 (95% CI 0.994–1.000).

In summary, out of 270 real-time measurements, the system misclassified 54 (20.0%) polyps during the first measurement and 24 (8.9%) polyps during the second measurement. Specifically, among the 189 real-time measurements for sizes ≥ 5 mm, the size was underestimated as < 5 mm for 3 (1.6%) polyps in the first measurement and 1 (0.5%) polyp in the second measurement. Furthermore, among the 216 real-time measurements for sizes < 10 mm, the size was overestimated as ≥ 10 mm for none of the first measurements and 1 (0.5%) of the second measurements. Among all polyp misclassifications, the medium-depth distance and within the center-FOV position exhibited the lowest misclassification rates, with zero polyps in both the first and second measurements. Additionally, the system exhibited terminal digit preferences of 5 mm and 10 mm for none of the first measurements and 3 (1.1%) of the second measurements. An intuitive representation is provided in Supplementary Fig. 9 and Supplementary Tables 7 and 11.

## Discussion

Based on 3D-CT reconstruction, we developed a real-time CAM system for measuring polyp sizes. The system demonstrated good performance in both various validation environments of 3D-MDS and real-time testing with a high-simulation colon/polyp model; the medium-depth distance and the position within the center FOV was most likely the optimal measurement locations, showing the best accuracy and reliability for polyp size measurement.

Previous studies have shown that the ability of endoscopists to estimate colorectal polyp size via traditional methods is very limited, with accuracies ranging from 54% to 65% [40, 41]. Visual estimation by endoscopists, the current de facto standard, suffers from well-documented inaccuracies and significant inter-observer variability, often influenced by terminal digit preference (e.g. clustering at 5 mm and 10 mm) [21–25]. Existing tool-assisted methods, while offering some improvement, introduce their own drawbacks. Methods relying on biopsy forceps, transparent caps, or laser probes require additional equipment, increase procedural time and cost, and their accuracy can be compromised by tangential positioning or the need for direct contact with the polyp [8–20]. Recent advancements in AI have shown promise in enhancing the accuracy and consistency of polyp size measurements. AI-assisted systems can potentially standardize measurements and reduce human error [26–30]. However, these AI-based systems still need colonoscopy reference media, including branches of the main blood vessels [26], auxiliary water jets [27], and biopsy forceps [28, 29] or are not measured in real time [26, 30], which does not enable intelligent measurement of colorectal polyp size. Therefore, accurate measurement of colorectal polyp size should not depend on endoscopic references, and a real-time automated measurement system that combines colonoscopy and AI is needed [42]. Our fully automated, real-time CAM system is designed to overcome these fundamental limitations. By integrating a polyp detection model and a depth-map prediction model based on monocular depth estimation, the system provides an objective, immediate measurement the moment a polyp is detected, without the need for any external references, auxiliary instruments, or manual intervention. This eliminates the subjectivity of visual estimation and the practical hurdles of tool-assisted methods. The system’s core innovation lies in its ability to generate a depth map from a standard 2D white-light image in real-time (within 28 ms per frame), calculate size based on the principle of triangle similarity, and apply fine-tuning for lens distortion and polyp thickness. This approach provides a pure software solution that leverages standard colonoscopy hardware, making it highly generalizable. By delivering consistent, reference-free measurements instantaneously, the CAM system has the strong potential to standardize polyp sizing across different endoscopists and institutions, thereby enhancing the reliability of clinical decisions regarding resection strategy and surveillance intervals.

One of the earliest reports on AI-assisted real-time automated measurement of polyp size was from the UK [43]. The team developed a method based on 3D reconstruction and implemented a convolutional neural network (CNN) model, achieving effective real-time classification of polyp size. However, its applicability was constrained by the requirement for large and robust datasets with ground-truth information. A recent exciting study from China evaluated an advanced deep learning-based system called ENDOANGEL-CPS for real-time measurement of colorectal polyp size [6, 44]. This system initially generates a real depth map for an endoscopic image and calculates polyp size based on the polyp depth distance and colonoscope lens parameters without the need for any other auxiliary instruments. Based on the testing of prerecorded images and videos, the accuracy of the system in measuring polyp size and predicting surveillance intervals was significantly greater than that of endoscopists. Notably, the CAM system further achieved good overall accuracy with mean ${\text{PE}}_{\text{Size}}$and CCC values of up to 8.3% (IQR 2.1%–13.5%) and 0.91 (95% CI 0.86–0.96), respectively, and high reliability between the two measurements with an ICC of 0.82 (0.77–0.85); these data were based on the real-time settings and accurate data of the ground-truth polyp size. Its optimal performance reached 3.3% (IQR 1.8%–3.5%), 0.99 (95% CI 0.87–0.99), and 0.999 (95% CI 0.994–1.000) at the medium-depth distance and within the center-FOV position, consistent with the endoscopists’ routine clinical observation of polyps. Thus, the CAM system has high applicability in routine clinical practice.

Current endoscopic products usually include only one lens; thus, it is difficult to obtain endoscopic images and the corresponding standard (ground truth) depth information of the image simultaneously. Although 3D scanning is often able to provide depth information, it can currently be used only *In vitro* [45]. Therefore, we utilized the 3D-CT reconstruction technique to create a ground truth dataset for training the depth-map model; this ensured that a large and accurate training dataset was used to predict the depth distance of the polyp, and was attained via 3D-MDS. This setting enabled the collection of a ground-truth dataset and verification of the performance of the depth-map prediction model not only in various colorectal environments, such as colorectal bends, folds, and relatively straight sections, but also under different lighting conditions. As a result, our system can be adapted to a wider range of environments to enable prospective real-time measurement in a real-world setting, indicating an improvement in polyp size measurement.

Compared with the subjective estimation by endoscopists, the principle of “triangle similarity” has recently been introduced to calculate polyp size and significantly enhances measurement accuracy [44]. However, several key factors, including the “fish-eye” effect and the “polyp-thickness” effect, still prevent the measurement from being optimal. Therefore, our system incorporates a fine-tuning procedure: when the depth distance is predicted, the CAM system incorporates the endoscopic lens FOV difference and the size of the polyp radius to achieve a more accurate estimation. Concurrently, we designed a high-simulation colon/polyp model and simulated polyps with diverse settings as close as possible to the human colorectum (Supplementary Video 2); this real-time testing further demonstrated the credibility of high accuracy for the CAM system.

Multiple studies have indicated that endoscopists frequently prefer the terminal digit, especially at terminal digit points of 10 mm and 5 mm [21, 22, 24, 25]. Our CAM system demonstrates minimal terminal digit preference (0.4% in 3D-MDS and 1.1% in the high-simulation colon/polyp model). By providing continuous, unbiased estimates of polyp size, the system reduces the clustering of measurements within specific intervals. A study involving 15 gastroenterologists revealed consistent underestimation of adenoma sizes [23], whereas another study demonstrated that endoscopists tended to overestimate colorectal polyp size [21]; these results indicated frequent occurrences of misclassification. In our study, the system had a high rate of misclassification in overestimating the size as ≥ 5 mm and underestimating the size as < 10 mm; this resulted in a significantly higher mean ${\text{PE}}_{\text{Size}}$ for the 5–10 mm polyp size category group in the two measurements than for the < 5 and ≥ 10 mm groups (*P *< 0.0001) (Supplemental Fig. 9). Specifically, in the 3D-MDS validation: out of 3,750 measurements, 227 RGB images of polyps with sizes ≥ 10 mm were underestimated as < 10 mm, a misclassification rate of 12.9% (227 of 1,757). This implies that for these cases, patients who should have closer surveillance (e.g. 3 years) might be incorrectly assigned to a longer interval (e.g. 5–10 years). Conversely, no polyps < 10 mm were overestimated as ≥ 10 mm. In the high-simulation model real-time testing: out of 270 first measurements, 3 polyps with sizes ≥ 5 mm were underestimated as < 5 mm, a rate of 1.6% (3 of 189). This could lead to patients being erroneously excluded from surveillance. Additionally, 1 polyp < 10 mm was overestimated as ≥ 10 mm in the second measurement, a rate of 0.5% (1 of 216), potentially causing unnecessary, more frequent colonoscopies. However, most misclassifications of polyp size by the system stemmed from measurements not conducted at the medium-depth distance or center-FOV position (Supplementary Table 7). These results highlight the importance of determining the optimal measurement location for the system since AI systems have difficulty to automatically identify the most suitable depth distance and FOV position for measuring polyp size [28]. This is reason that our design had near, medium, and far depth distances and 9 FOV positions to test the system’s performance. To mitigate measurement bias around critical size thresholds (e.g. 5 mm and 10 mm), we plan to enhance the CAM system through the following improvements: algorithm optimization to reduce measurement errors near these key decision thresholds; incorporation of a confidence metric into the system output, alerting endoscopists to confirm with auxiliary tools or repeat measurements when results approach clinical decision thresholds; and development of an adaptive calibration module, enabling the system to dynamically adjust measurement strategies based on real-time image characteristics.

Compared with previous studies, our study has several strengths. First, in our study on AI-assisted real-time automated polyp size measurement, we were the first to conduct prospective real-time testing of a high-simulation colon/polyp model, achieving favorable results. Based on the prospective real-time testing of the high-simulation colon/polyp model, which could provide the most accurate reference for measurement, the current study took a significant step toward integrating AI into routine endoscopic practices for measuring polyp size. Second, our study used 3D-CT reconstruction to create a comprehensive and accurate depth-map dataset for training and testing the depth-map prediction model in 3D-MDS with various complex environments. This approach addresses the challenge of acquiring extensive high-quality depth data in real-world settings and provides a solid foundation for adapting a CAM system to diverse environments, thereby facilitating prospective real-time measurement. Third, our study was designed to test the CAM system performance with near-, medium-, and far-depth distances and 9 FOV positions. We demonstrated the good performance of the system in each depth distance and FOV position and determined the optimal measurement location. The present study lays the groundwork for the next steps in preclinical and clinical trials.

Nonetheless, this study has several limitations. First, we tested the real-time depth distance measurement with graduated biopsy forceps only at the center-FOV position. This method is not highly accurate and other FOV positions cannot be estimated with graduated biopsy forceps; however, it is likely the easiest and most reliable method to obtain the ground truth for depth distance, and the results indicated that the graduated biopsy forceps and the system show good agreement in real-time depth distance measurement. Second, the system has only been validated on 3D-MDS and high-simulation colon/polyp model; however, unlike real polyps in colonoscopy practice, virtual and simulated polyps, rather than real colorectal polyps, can provide highly accurate ground truths for validation. Our validation in real polyps or clinical trials is in progress and will be used to assess the reliability of the CAM system in colonoscopy practice. Our plan for clinical translation to address this limitation includes pilot study based on colorectal polyp videos, single-center, parallel, single-blind clinical trials, multi-center clinical trials, long-term outcomes like CRC incidence reduction, and regulatory approval (National Medical Products Administration in China and Food and Drug Administration in the USA) and implementation. Third, data on endoscopists’ estimation of polyp size are lacking; however, the system is still in the development phase, and the next series of trials will be conducted by endoscopists using subjective assessment, auxiliary tools, and autonomous AI measurement as a reference for AI-assisted human measurement [34]. Fourth, the ${\text{PE}}_{\text{Size}}$ is slightly greater in the morphology subgroup of flat polyps (0-IIa); this is an inherent issue with flat lesions, particularly when measurements are not taken at medium-depth distance or at the center-FOV position. Fifth, this preliminary study uses a sample size sufficient for assessing technical feasibility; the definitive large-scale trial will be powered based on a formal calculation. Sixth, the simulated polyps in our model were designed based on general morphological characteristics and did not specifically replicate the unique and subtle features of sessile serrated lesions, such as their cloud-like surface, mucus caps, or irregular borders. Similarly, other non-polypoid lesions like laterally spreading tumors or depressed types were not included in this validation phase; future validation work must include prospective clinical trials involving a wide spectrum of real colorectal lesions, with a specific focus on challenging subtypes such as sessile serrated lesions and other non-polypoid lesions. Finally, the system was validated exclusively on real-time white light images; future considerations may include incorporating narrowband imaging.

## Conclusions

We developed and preliminarily validated a real-time CAM system for polyp size measurement based on 3D-CT reconstruction. This innovative system has the potential to increase the accuracy and reliability of colorectal polyp size measurement, particularly at the medium-depth distance (15–35 mm) and within the center-FOV position, which is likely the optimal measurement location.

## Supplementary Material

goag041_Supplementary_Data

## Supplementary material

**Video 1** Overview of the study and the CAM system.

**Video 2** Overview of the high-simulation colon/polyp model.
