# Supplementary material for: Development and validation of a real-time computer-aided measuring system for colorectal polyp size (with video)
Source: Gastroenterol Rep (Oxf). 2026 May 12;14:goag041. doi: 10.1093/gastro/goag041 (PMC13163181; doi:10.1093/gastro/goag041)
Supplement: goag041_Supplementary_Data [file goag041_supplementary_data.zip › Supplementary__Figures_ final version.docx]

**Supplementary Figures**


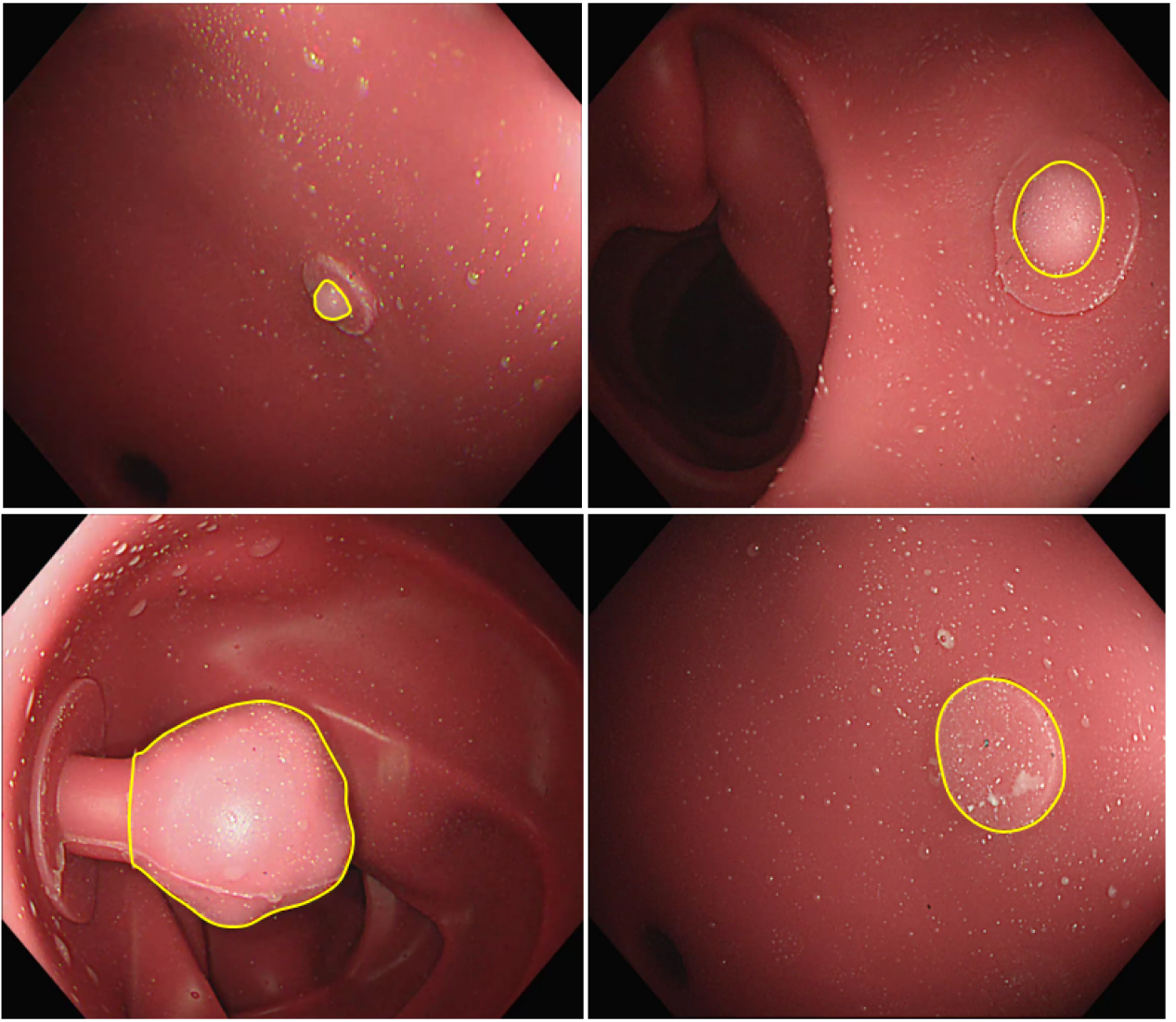


**Supplementary Figure 1.** Representative images of polyp detection model in the high-simulation colon/polyp model.


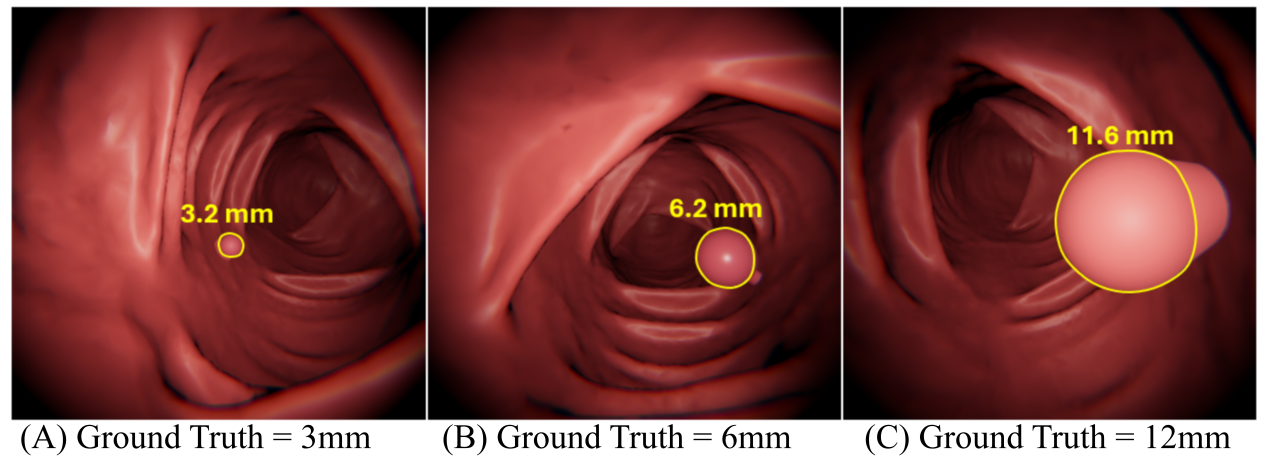


**Supplementary Figure 2.** Representative images of polyp size measurement in validation of digitized three-dimensional model development setting. The ground truth sizes are 3mm, 6mm and 12mm for (A), (B) and (C), respectively, and the system output the polyp sizes are 3.2mm, 6.2mm, and 11.6mm, respectively.


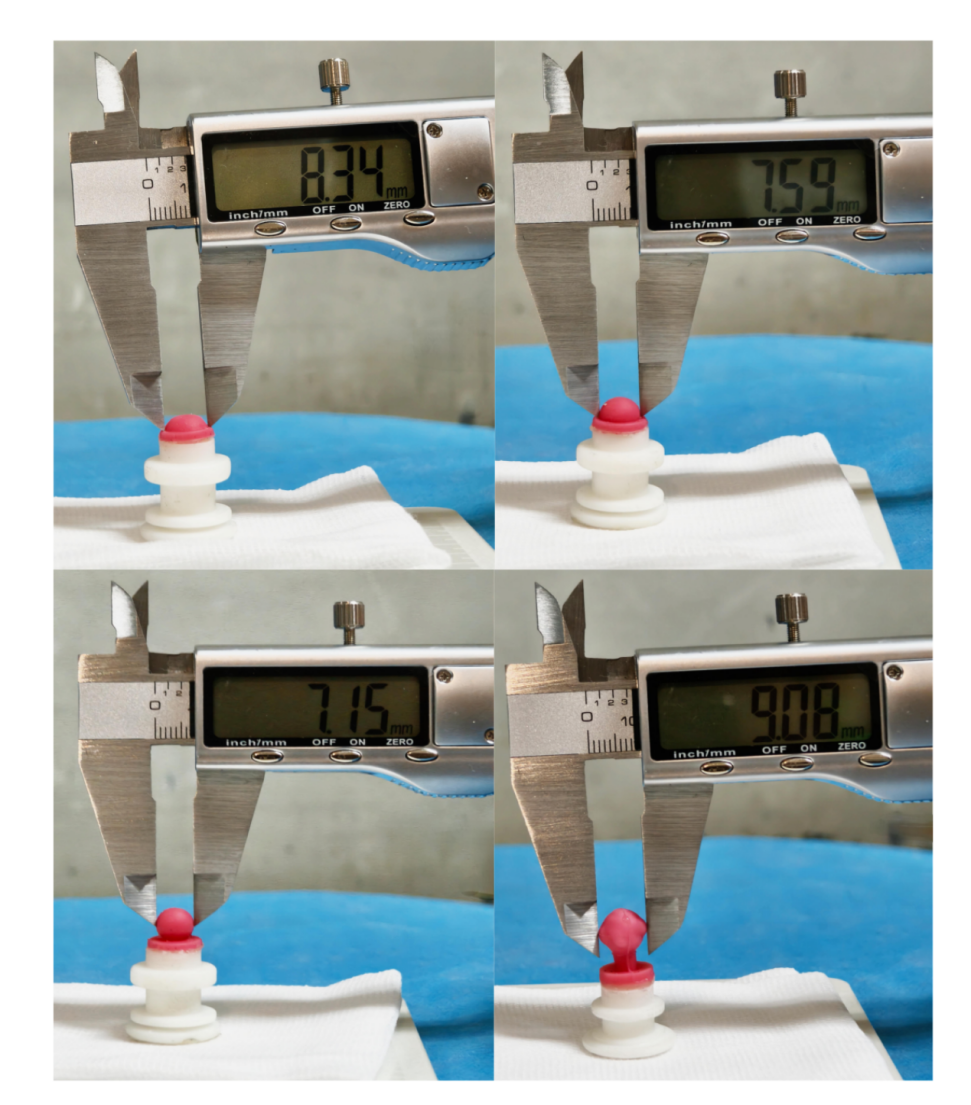


**Supplementary Figure 3.** Representative images of simulated polyp size measurement with vernier calipers.


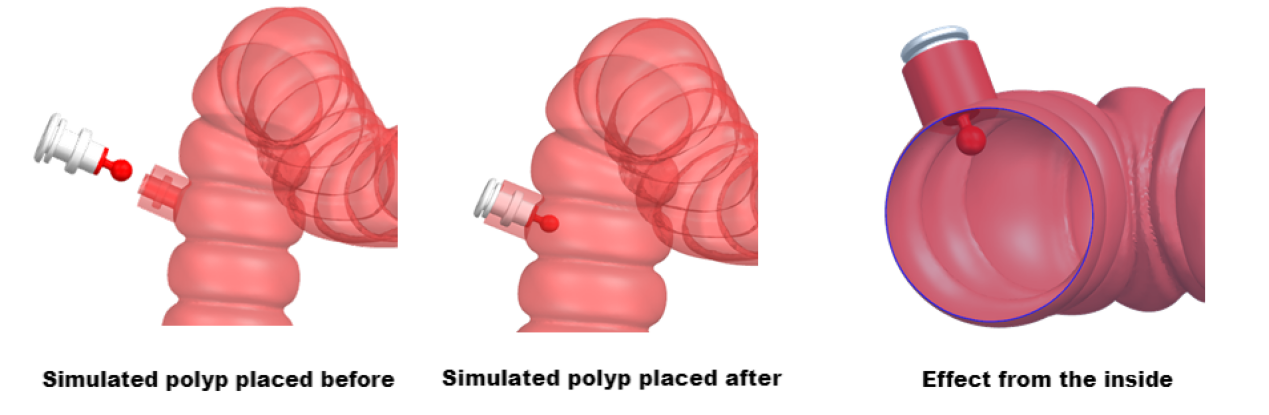


**Supplementary Figure 4.** Schematic diagram of simulated polyp replacement of the high-simulation colon/polyp model.


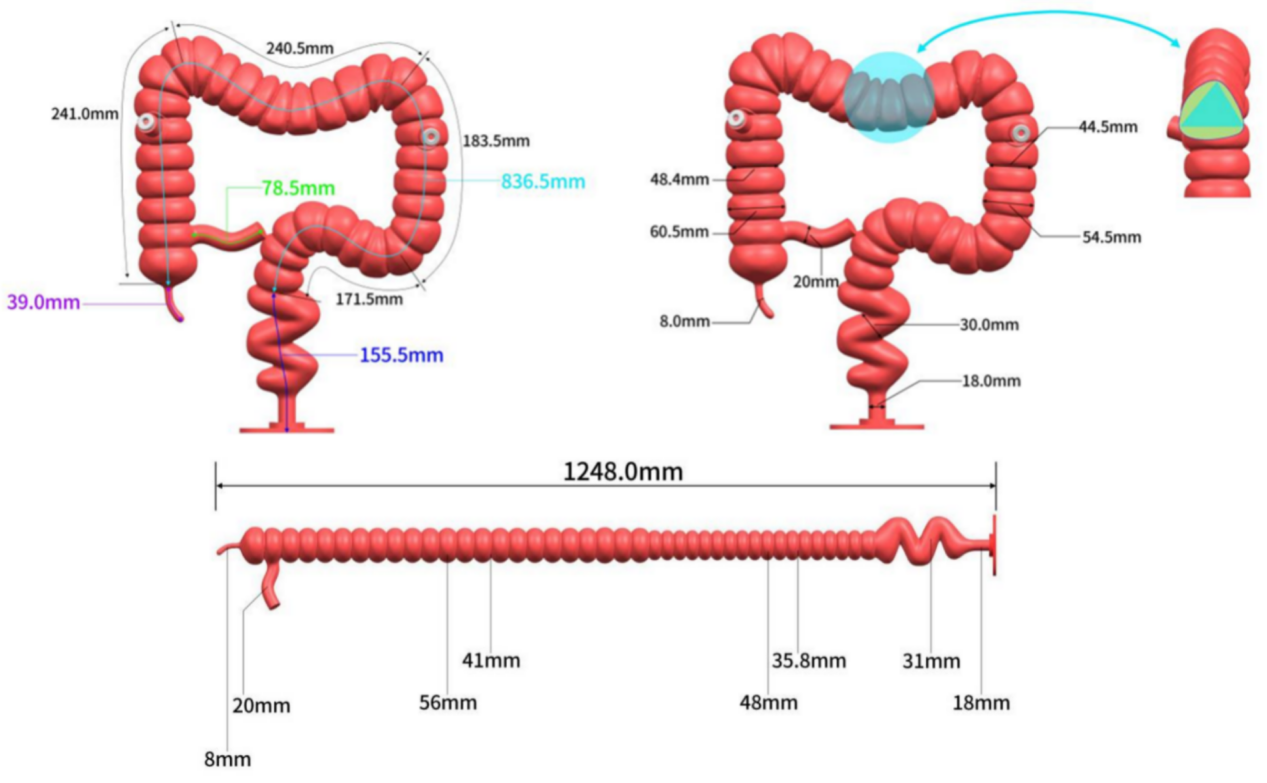


**Supplementary Figure 5.** Schematic diagram of colorectal parameters of the high-simulation colon/polyp model.


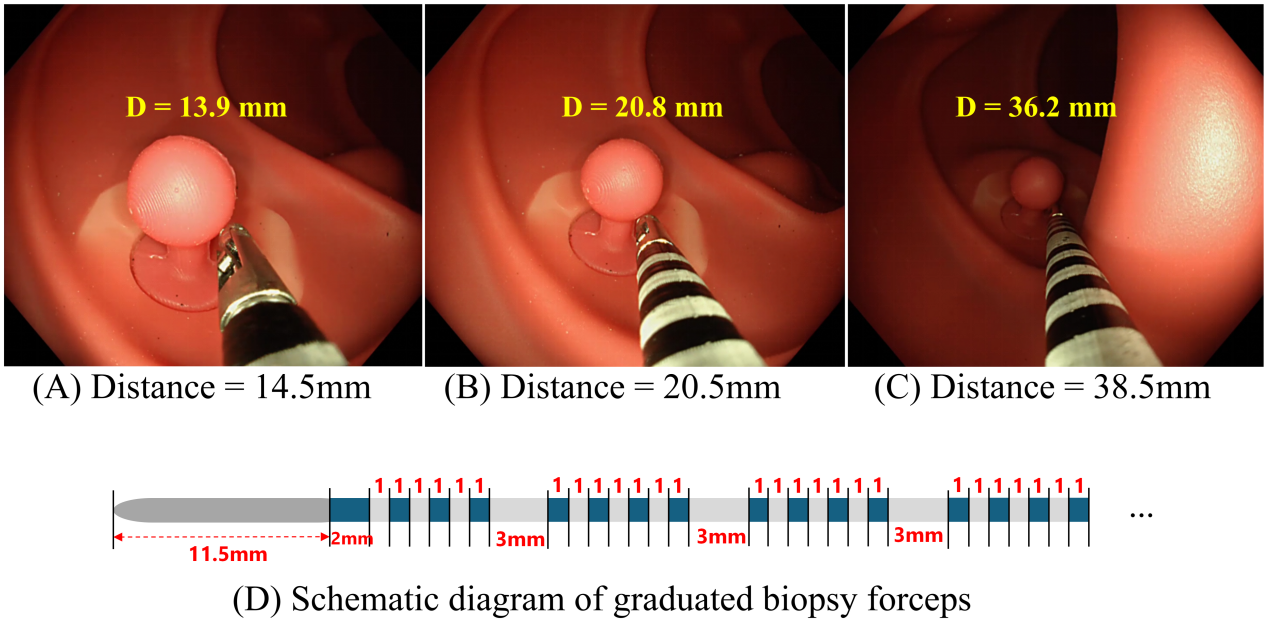


**Supplementary Figure 6.** Representative images of graduated biopsy forceps and computer-aided measuring system for real-time depth distance measurement in the high-simulation colon/polyp model. The depth distances estimated by the graduated biopsy forceps are 14.5mm, 20.5mm, and 38.5mm for (A), (B), and (C), respectively, and the system output the depth distances are 13.9mm, 20.8mm, and 36.2mm, respectively. (D) The parameters of graduated biopsy forceps.


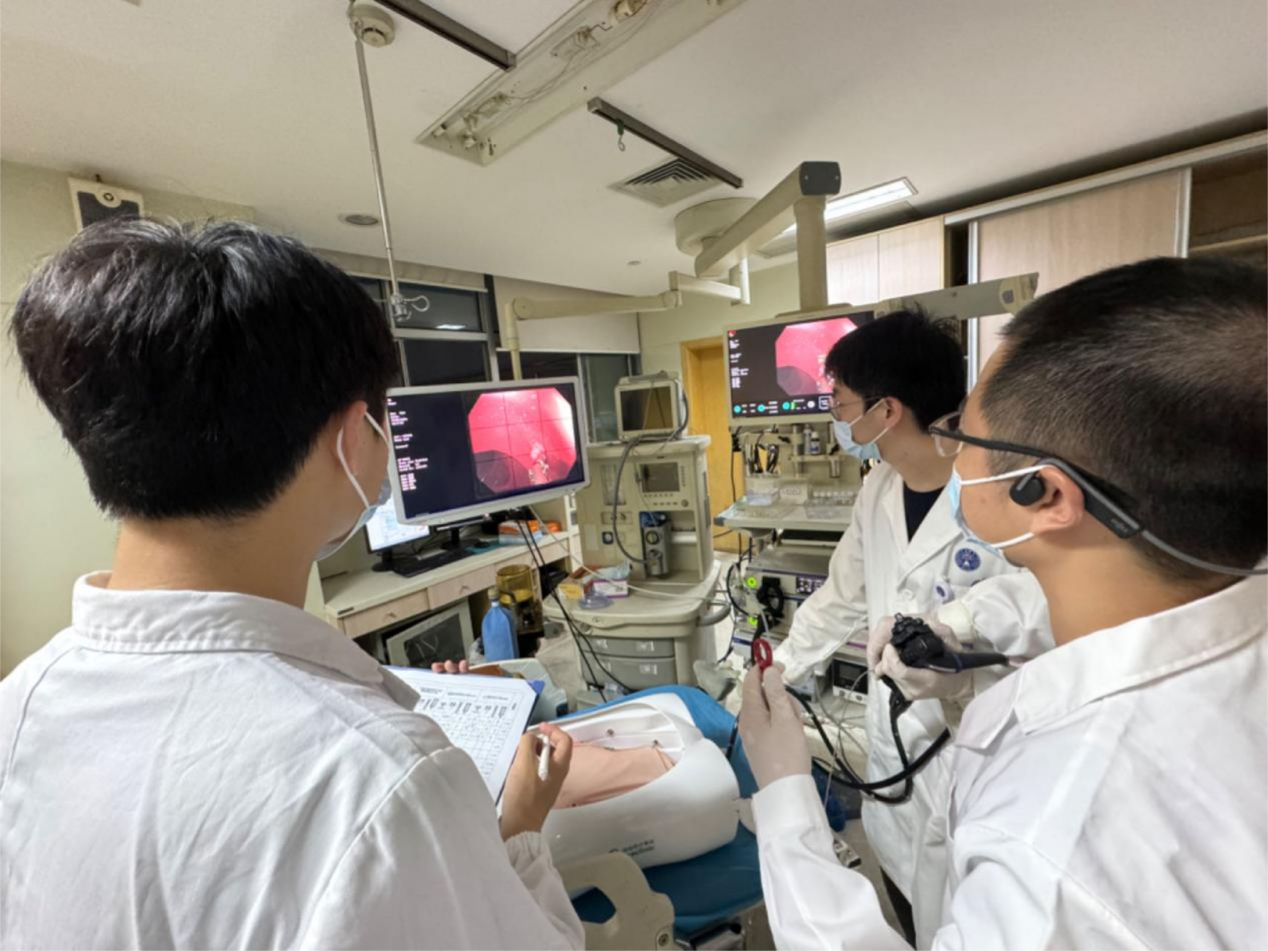


**Supplementary Figure 7.** Representative images of the study environment during real-time measurement.


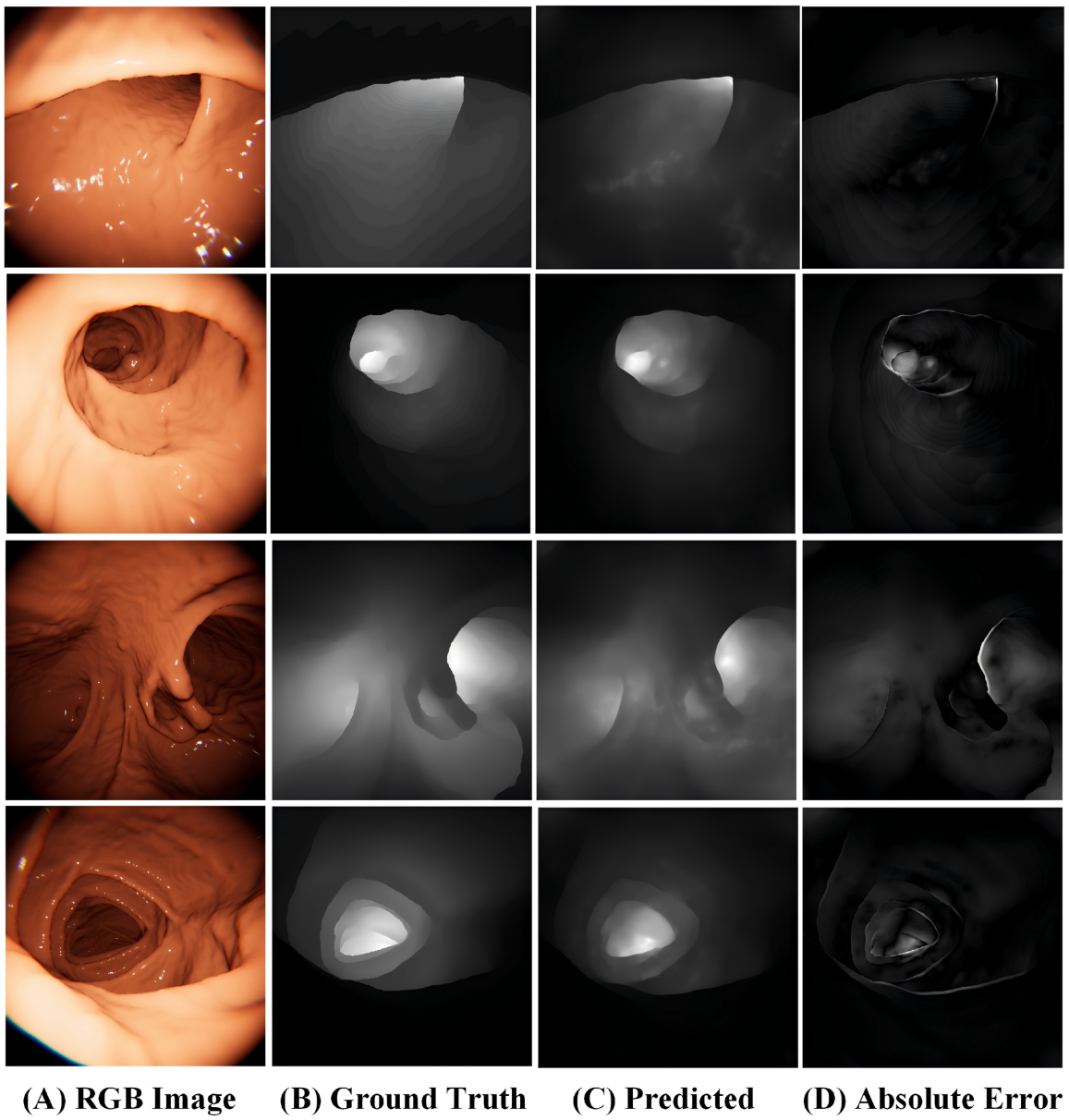


**Supplementary Figure 8.** Results of depth-map prediction. (A), (B), (C) and (D) presented the RGB (red-green-blue) images, ground truth depth map, predicted depth map, and absolute errors.


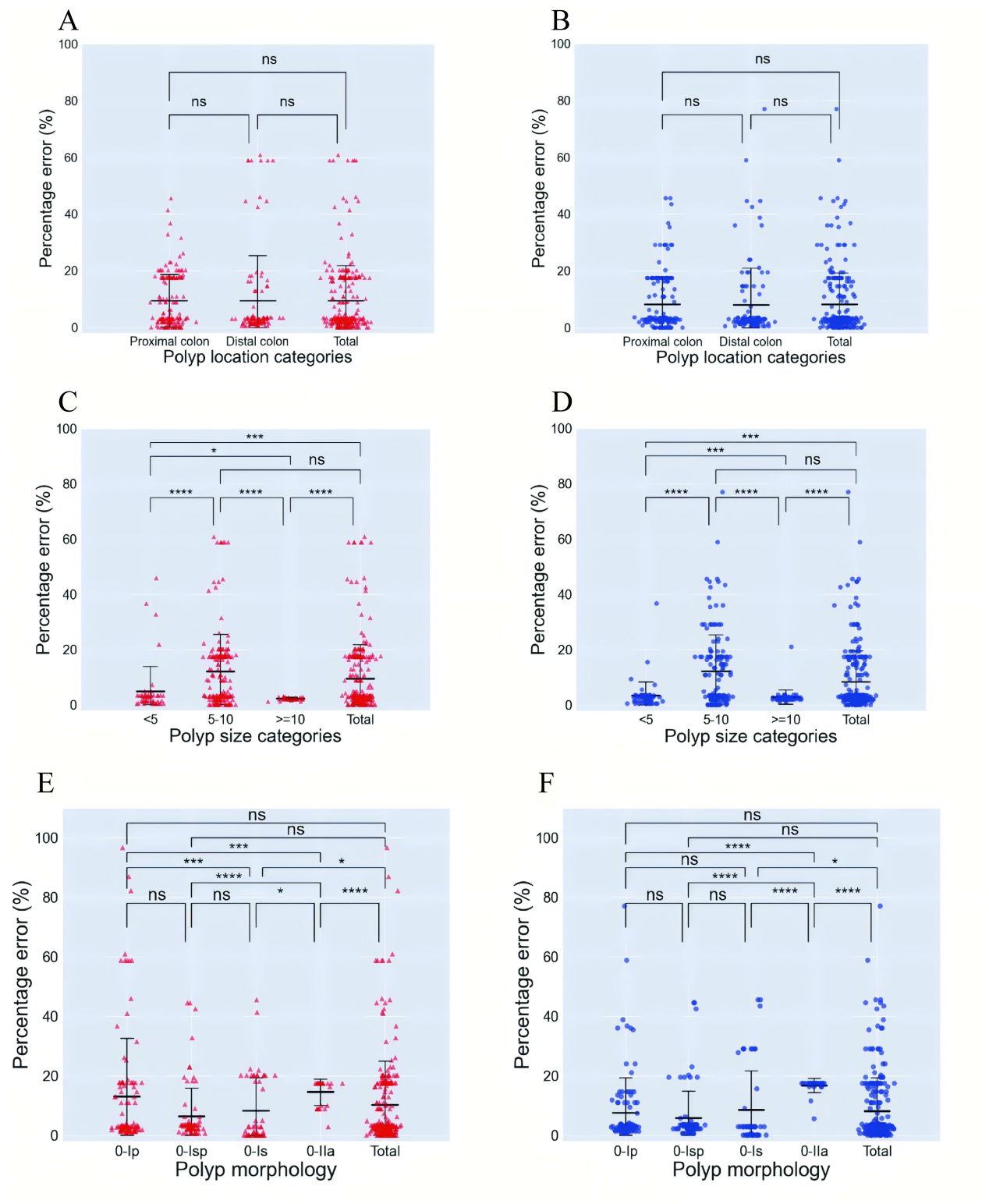


**Supplementary Figure 9.** Real-time performance of polyp size measurement in the high-simulation colon/polyp model according to different subgroups. **(**A), (C) and (E) The performance of total 1st measurement. (B), (D) and (F) The performance of total 2nd measurement. (A) and (B) Percentage errors in proximal colon and distal colon. (C) and (D) Percentage errors in polyp size categories according to the cut-off of 5 and 10 mm. (E) and (F) Percentage errors in different polyp morphology. ns, not significant. * *P* < 0.05; *** *P* < 0.001; **** *P* < 0.0001.


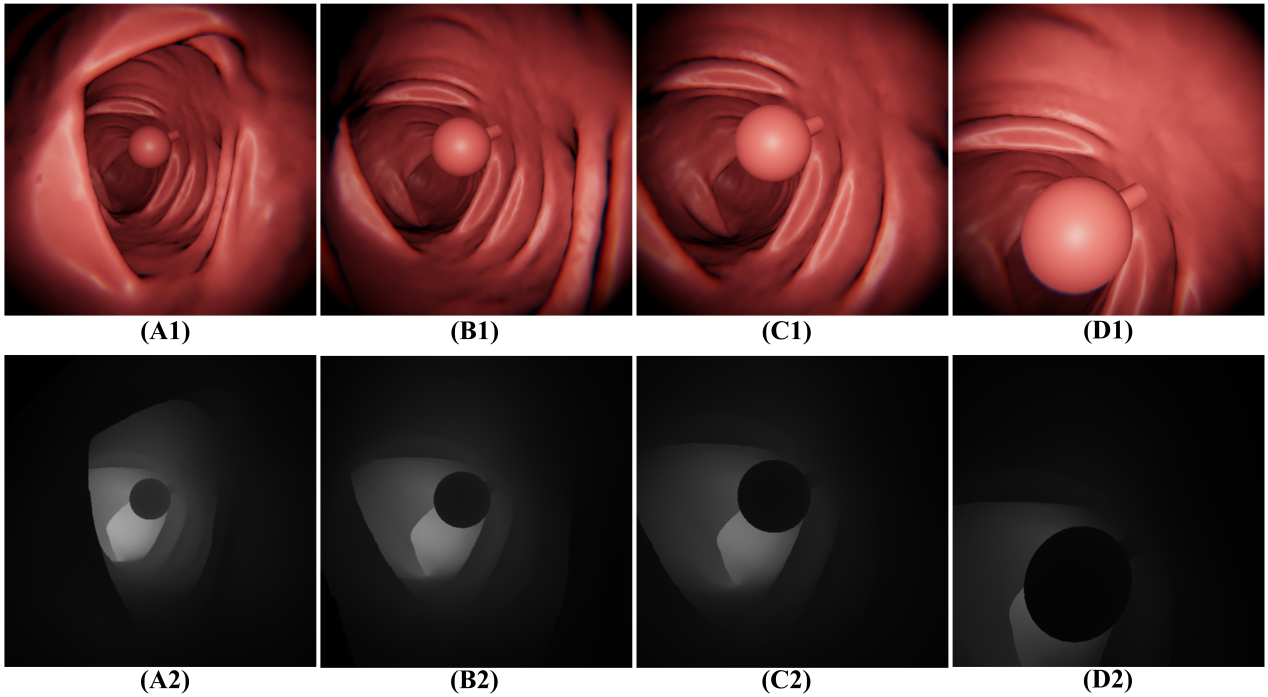


**Supplementary Figure 10.** Representative images of the collected 2D colorectal images and their corresponding 2D ground truth depth-map images.


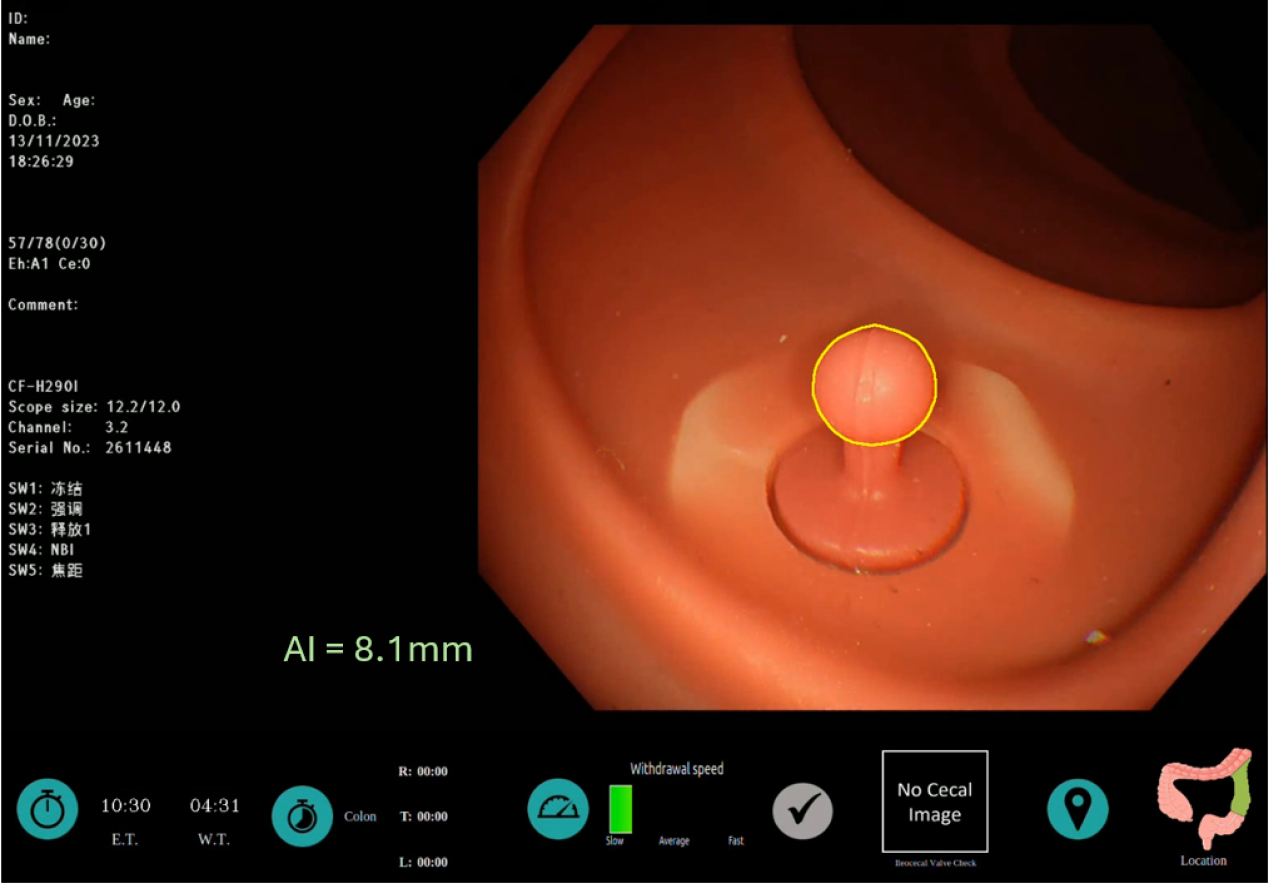


**Supplementary Figure 11.** A representative image of the real-time output of the CAM system in the high-simulation colon/polyp model. CAM, computer-aided measuring.


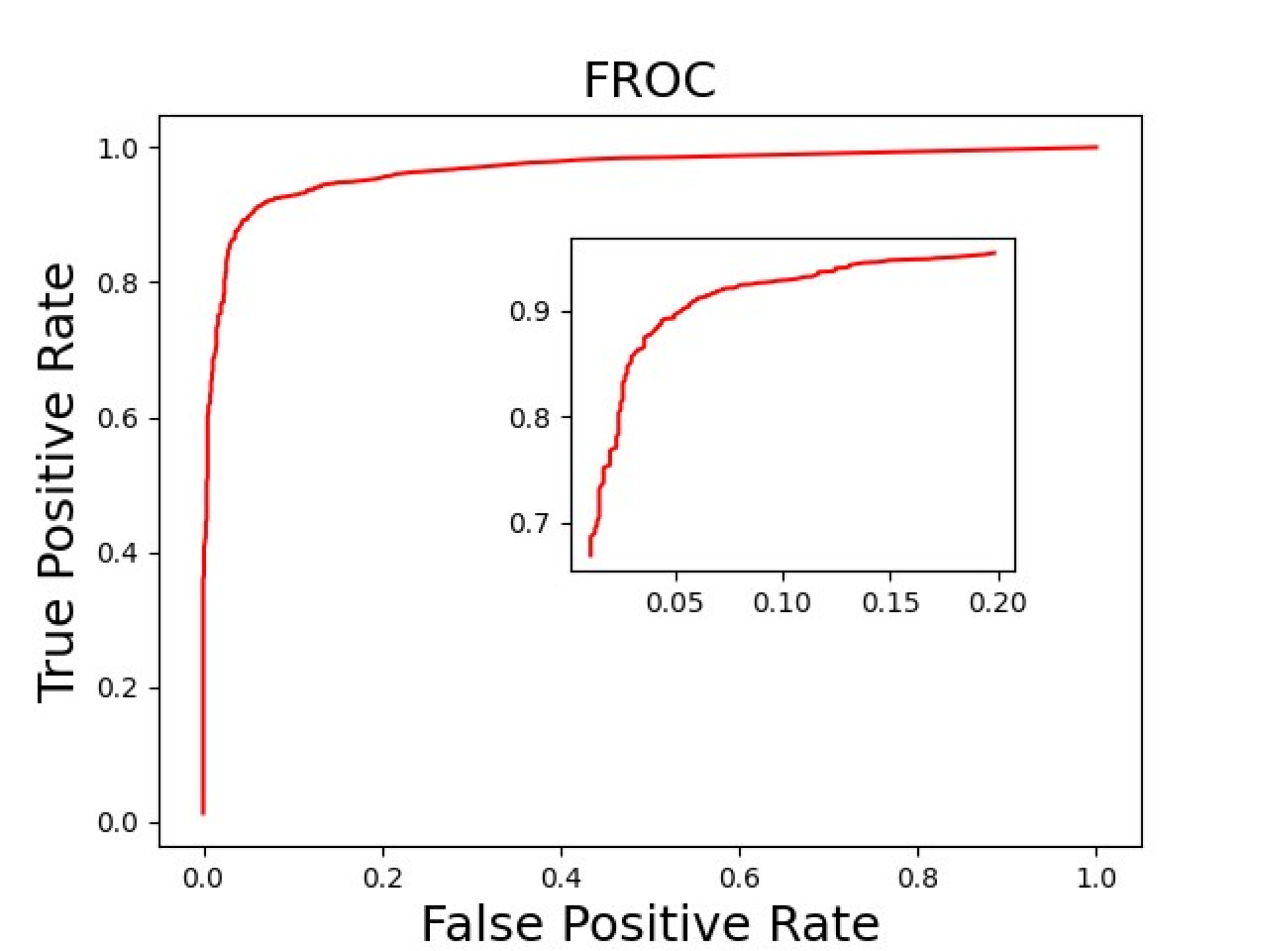


**Supplementary Figure 12.** The free-receiver operating characteristic curve of the polyp detection model. FROC, free-receiver operating characteristic.
