# Supplementary material for: Development and validation of a real-time computer-aided measuring system for colorectal polyp size (with video)
Source: Gastroenterol Rep (Oxf). 2026 May 12;14:goag041. doi: 10.1093/gastro/goag041 (PMC13163181; doi:10.1093/gastro/goag041)
Supplement: goag041_Supplementary_Data [file goag041_supplementary_data.zip › Supplementary_material_ final version.docx]

**Supplementary Material**

**Methods**

***Dataset***

**Dataset for polyp detection model**

Obtaining high-quality and efficient data for deep learning-based solutions presents a significant challenge. In medical applications, this challenge is further compounded by two major issues. First, obtaining patient consent is essential to address data protection concerns. Second, the task of annotating data requires the expertise of medical professionals, which is both time-consuming and complex. Therefore, to develop a polyp detection model, we use publicly available data from the Internet along with data collected from Tri-Service General Hospital and Show Chwan Memorial Hospital. **Figure 1** shows a flowchart of image preparation for training the polyp detection model. We use nine polyp image datasets including Kvasir-SEG [1], CVC-ClinicDB [2], CVC-ColonDB [3], CVC-300 [4], ETIS‐LaribPolypDB [5], EndoCV2022 [6], EDD2020 [7], and our collected dataset for training and validation, and use PICCOLO [8] for testing.

We combine the publicly available data with our collected data to obtain a total dataset of 20,058 images. All data comprise images and their bounding boxes. An intuitive representation is shown in **Figure 2A1** and **Supplementary Table 12**.

**Dataset of depth-map prediction model**

The depth-map prediction model is used to predict the depth information of each pixel in an image. Therefore, the training dataset must contain depth information for each pixel of each input image. Model training requires annotated depth data, which usually correspond to the input images. Annotations can be generated from three-dimensional (3D) scanners, stereo cameras, depth sensors, or other depth information sources.

In a digitized 3D model development setting (3D-MDS), we remotely control the movement of a virtual camera to simultaneously collect red–green–blue endoscopic colorectal images and corresponding ground truth depth-map images. The parameters of the virtual camera are generated with reference to those of an Olympus endoscope.

**Figure 2A2 and 2D1** show the 3D-MDS in the Unity environment. To increase the diversity of depth data, we created 96 combinations in the digitized virtual 3D colorectal model based on different polyp morphologies, polyp sizes, and light brightness levels. In each combination, we collected 210 samples at different distances and angles, resulting in a total of 20,000 2D colorectal images and 2D ground truth depth maps. **Supplementary Figure 10** shows the 2D colorectal images and their corresponding 2D ground truth depth-map samples.

***Polyp detection model***

We used a deep hybrid neural network (DHNN) [9] as the polyp detection model in this study. The deep hybrid neural network provides a novel concept by simultaneously adopting linear and nonlinear operators as well as combining convolutional and mathematical morphological layers. The model generates convolutional and morphological backbones in parallel to extract linear and nonlinear features, which are then combined for further analysis (**Figure 2B1**).

All images were resized to 640×480 pixels to match the polyp detection model. To increase the diversity of the training data, we employed data augmentation techniques, such as horizontal/vertical flipping, height/width shifting, brightness variation, noise addition, and contrast enhancement. We trained the model for 100 epochs and selected the model with the highest intersection over union as the final polyp detection model.

The polyp detection model is central to polyp identification and localization. It performs real-time analysis of endoscopic images, effectively extracts features from complex biological images, and accurately detects polyps across multiple scales and viewpoints. The model’s performance directly impacts the diagnostic accuracy and clinical utility of the CAM system, making it a crucial component in polyp size measurement.

***Depth-map prediction model***

The depth-map prediction model primarily comprises an encoder and a decoder. The encoder is responsible for feature extraction, and we use ResNet-50, an open-source model proposed by He *et al.* in 2016 [10]. The name ResNet-50 originates from the network structure, which has 50 convolutional layers and includes residual blocks. These residual blocks help address the vanishing and exploding gradient issues in deep network training. ResNet-50 incorporates residual blocks, each containing multiple convolutional layers and skip connections, which facilitate information flow throughout the network. The decoder is responsible for depth-map prediction and output, and we use DenseDepth [11]. DenseDepth comprises a 2× bilinear upsampling step and two standard convolutional layers, and it performs very well in experiments. Specifically, in the depth estimation network, the decoder works in conjunction with the encoder to generate high-resolution depth maps by converting red–green–blue images into feature vectors (**Figure 2B2**).

The depth-map prediction model was pretrained with ImageNet. All images were resized to 640×480 pixels to match the depth-map prediction model. Similarly, to increase the diversity of the training data, we employed data augmentation techniques, including horizontal/vertical flipping, height/width shifting, noise addition, and contrast enhancement. We trained the model for 100 epochs and selected the model with the minimum average depth error as the final depth-map prediction model.

In the CAM system, the depth-map prediction model and polyp detection model are integrated to create a synergistic mechanism. The polyp detection model initially identifies and localizes a polyp, providing its 2D location information. The depth-map prediction model then estimates the polyp depth distance in 3D space. This integrated approach enhances the accuracy of polyp localization and offers a more precise polyp size measurement by combining depth information. The synergy between these two models facilitates efficient and accurate polyp size measurement, thereby supporting more precise clinical diagnosis and treatment planning.

***Definition of autonomous AI measurement***

Once a polyp is detected in an image, the CAM system initiates the depth-map prediction model to predict the depth distance and calculate the polyp size. The tracking algorithm works synchronously, continuously tracking the polyp in subsequent images. After accumulating 30 frames of polyp images, the CAM system compiles their depth distances or sizes and uses the median as the autonomous AI polyp size estimation. An intuitive representation is shown in **Supplementary Figure 11**.

***Real-time testing in a high-simulation colon/polyp model***

A high-simulation colon/polyp model and simulated polyps were proposed by our team and constructed by Preclinic Medtech (Shanghai) Co., Ltd. in P. R. China. In addition, our team holds several patents in P. R. China as the primary applicant for this model and has initiated a patent application in the United States for the associated invention.

***Endoscopy, graduated biopsy forceps and digital vernier caliper***

The colonoscopes used in this study are Olympus CF-H290I and CF-HQ290I (Olympus Ltd., Tokyo, Japan), graduated biopsy forceps (HEALCARE Medical Instrument Co., Ltd., Jiangyin, Jiangsu, P. R. China), digital vernier caliper (MYTEC Tools Co., Ltd., Jiangyin, Jiangsu, P. R. China).

***Parameters of the CAM system***

The hardware parameters are as follows: the CPU version is an Intel Core i9-11900KF processor with base and maximum turbo frequencies of 3.50 and 5.30 GHz, respectively.

The GPU is an NVIDIA GeForce RTX 3090 (memory size: 24 GB, memory bandwidth: 384 bits). Architecture: Ampere, CUDA Cores: 10496.

Running time: The polyp detection and depth-map prediction models take 9 and 12 ms, respectively, to obtain a polyp and depth-map, and the CAM system takes 7 ms to obtain video and screenshot images. Overall, it takes 28 ms to process an input image.

***Statistical analysis***

**Statistical analysis of the polyp detection model**

Let true positive (TP) denote the predicted bounding box that falls on the ground truth of a polyp; false positive (FP) denote the predicted bounding box that falls outside the ground truth; false negative (FN) indicate that no predicted bounding box is present despite at least one polyp existing in the frame; true negative (TN) indicate that no polyps are detected in images without polyps.

Precision indicates how many estimated positives are TPs. Thus, it is a key metric accounting for the cost of FPs and is calculated as follows:

$$\text{Precision}\text{ }\text{=}\text{ }\frac{\text{TP}}{\text{TP}\text{ }\text{+}\text{ }\text{FP}}\text{ (}\text{1}\text{)}$$

Recall indicates how many TPs are identified from all actual positives. It is sometimes referred to as sensitivity and is calculated as follows:

$$\text{Recall}\text{ }\text{=}\text{ }\text{Sensitivity}\text{ }\text{=}\text{ }\frac{\text{TP}}{\text{TP}\text{ }\text{+}\text{ }\text{FN}}\text{ (}\text{2}\text{)}$$

Specificity indicates how many estimated negatives are TNs and is calculated as follows:

$$\text{Specificity}\text{ }\text{=}\text{ }\frac{\text{TN}}{\text{TN}\text{ }\text{+}\text{ }\text{FP}}\text{ }\left( \text{3} \right)$$

The F1 score represents the harmonic mean of the precision and recall metrics. It is particularly useful when a balance between precision and recall is needed. It can be expressed as follows:

$$\text{F}\text{1}\text{ }\text{=}\text{ }\frac{\text{2}\text{ }\text{∗}\text{Precision}\text{ }\text{∗}\text{Recall}}{\text{Precision}\text{ }\text{+}\text{ }\text{Recall}}\text{ }\left( \text{4} \right)$$

The area under the curve (AUC) represents the area under the free-receiver operating characteristic (FROC) curve. It measures the overall performance of a binary classification model. Since both the true positive rate (TPR) and false positive rate (FPR) range between 0 and 1, AUC always lies between 0 and 1. A higher AUC indicates better model performance.

**Results**

***Performance evaluation***

The testing dataset included 3,433 images containing polyps and 1,005 images that did not contain polyps. All polyps were confirmed histologically after biopsy. **Figure 2B1** and **Supplementary Figure 12** show the FROC curves of the polyp detection model, with an AUC of 0.97.

**Supplementary Table 13** shows the results of polyp detection on the PICCOLO dataset. With the sensitivity set to 90%, the specificity and FP count were 94.63% and 54, respectively. With the FPR set to 0.1, the sensitivity and FN count were 92.92% and 243, respectively.

**Supplementary Table 14** presents the detailed performance results for subgroups of polyps according to histology, morphology, and size. In terms of histology, adenoma polyp images were predicted more accurately than average, with the highest F1 score of 0.956. In terms of morphology, there are significant variations. The F1 score for flat polyp images (0.906) was significantly lower than the average. However, images of protruded polyps were predicted more accurately than the average, achieving the highest F1 score of 0.951. Smaller polyps seem to be more difficult to detect than larger polyps (F1 scores of 0.941 and 0.850, respectively).

**References**

**[dataset]** 1. Debesh J, Smedsrud HP, Riegler M, *et al.* Kvasir-SEG: A Segmented Polyp Dataset. In Proceedings of the trinational conference on Multimedia Modeling. Polyps Data. 2020. <https://datasets.simula.no/kvasir-seg/>

**[dataset]** 2. Bernal J, Sanchez FJ, Fernandez-Esparrach G, *et al.* WM-DOVA maps for accurate polyp highlighting in colonoscopy: Validation vs. saliency maps from physicians. Polyps Data. 2015. <https://paperswithcode.com/dataset/cvc-clinicdb>

**[dataset]** 3. Bernal J, Tajbakhsh N, Sanchez FJ, *et al.* Comparative validation of polyp detection methods in video colonoscopy: results from the miccai 2015 endoscopic vision challenge. Polyps Data. 2017. <http://vi.cvc.uab.es/colon-qa/cvccolondb/>

**[dataset]** 4. Vázquez D, Bernal J, Sánchez FJ, *et al.* A benchmark for endoluminal scene segmentation of colonoscopy images. Polyps Data. 2017. <https://www.kaggle.com/datasets/nourabentaher/cvc-300>

**[dataset]** 5. Silva JS, Histace A, Romain O, *et al.* Toward embedded detection of polyps in WCE images for early diagnosis of colorectal cancer. Polyps Data. 2014. <https://www.kaggle.com/datasets/mahmudulhasantasin/etis-laribpolypdb>

**[dataset]** 6. Sharib Ali NG (2022) Endoscopic computer vision challenges 2.0. Polyps Data. 2022. <https://endocv2022.grand-challenge.org/>

**[dataset]** 7. Ali S, Ghatwary N, Braden B, *et al.* Endoscopy disease detection challenge 2020. Polyps Data. 2020. <https://datasetninja.com/edd2020>

**[dataset]** 8. Sánchez-Peralta LF, Pagador JB, Picón A, *et al.* PICCOLO white-light and narrow-band imaging colonoscopy dataset: a performance comparative of models and datasets. Polyps Data. 2020. <https://www.biobancovasco.bioef.eus/en/Sample-and-data-catalog/Databases/PD178-PICCOLO-EN.html>

9. Wu Y, Shih FY, Wang C, *et al.* The deep hybrid neural network and an application on polyp detection. Intern J Pattern Recognit Artif Intell. 2024;38.

10. He K, Zhang X, Ren S, *et al.* Deep residual learning for image recognition. IEEE Conference on Computer Vision and Pattern Recognition, Las Vegas. 2016.

11. Alhashim I, Wonka P. High quality monocular depth estimation via transfer learning. Ithaca: Cornell University Library, arXiv.org. 2019.
