# Supplementary material for: Development and validation of a real-time computer-aided measuring system for colorectal polyp size (with video)
Source: Gastroenterol Rep (Oxf). 2026 May 12;14:goag041. doi: 10.1093/gastro/goag041 (PMC13163181; doi:10.1093/gastro/goag041)
Supplement: goag041_Supplementary_Data [file goag041_supplementary_data.zip › Supplementary_Tables_ final version.docx]

**Supplementary Tables**

**Supplementary Table 1.** Results of polyp size measurement in the digitized three-dimensional model development setting.

| Ground Truth (mm) | CAM system measurement, mm, mean (IQR) | ${\text{M}\text{E}}_{\text{Size}}$, mm,  mean (IQR) | $\text{PE}_{\text{Size}}$, %,  mean (IQR) |
| --- | --- | --- | --- |
| 2.0 | 2.3 (2.0, 2.6) | 0.3 (0.1–0.6) | 17.1 (7.0–27.6) |
| 3.0 | 3.4 (3.0, 3.7) | 0.4 (0.1–0.7) | 13.4 (4.3–22.4) |
| 4.0 | 4.4 (4.1, 4.7) | 0.4 (0.1–0.7) | 10.6 (3.4–17.5) |
| 5.0 | 5.4 (5.1, 5.7) | 0.4 (0.1–0.7) | 8.5 (3.0–13.5) |
| 6.0 | 6.4 (6.0, 6.7) | 0.5 (0.2–0.7) | 7.7 (3.0–11.9) |
| 7.0 | 7.2 (6.9, 7.5) | 0.3 (0.1–0.5) | 4.6 (1.9– 7.4) |
| 8.0 | 8.2 (7.9, 8.5) | 0.3 (0.2–0.5) | 4.1 (1.9–6.0) |
| 9.0 | 9.1 (8.7, 9.5) | 0.4 (0.2–0.6) | 4.4 (2.1–6.6) |
| 10.0 | 9.7 (9.3, 10.1) | 0.4 (0.2–0.7) | 4.2 (1.8–6.6) |
| 11.0 | 10.5 (10.0, 10.9) | 0.6 (0.3–1.0) | 5.6 (2.3–8.8) |
| 12.0 | 11.4 (11.0, 11.7) | 0.6 (0.3–1.0) | 5.3 (2.7–8.4) |
| 13.0 | 12.2 (11.8, 12.6) | 0.8 (0.4–1.2) | 6.2 (3.1–9.1) |
| 14.0 | 12.8 (12.3, 13.4) | 1.2 (0.6–1.7) | 8.3 (4.4–12.0) |
| 15.0 | 13.7 (13.2, 14.2) | 1.3 (0.8–1.8) | 8.5 (5.1–12.2) |
| 16.0 | 14.3 (13.7, 14.9) | 1.7 (1.1–2.3) | 10.4 (6.8–14.5) |
| Average | 8.7 (5.4, 12.2) | 0.6 (0.4–0.8) | 7.9 (4.6–10.4) |

IQR, interquartile range; CAM, computer-aided measuring; ${\text{M}\text{E}}_{\text{Size}}$, measurement error of polyp size; $\text{PE}_{\text{Size}}$, percentage error of polyp size; CCC, concordance correlation coefficient.

**Supplementary Table 2.** Polyp size misclassification according to the cut-off of 5 and 10 mm in the digitized three-dimensional model development setting.

| Polyp size misclassifications | *n* (%) |
| --- | --- |
| ≥ 5mm misclassified as < 5mm | 39/3,012 (1.3) |
| < 5mm misclassified as ≥ 5mm | 5/738 (0.7) |
| ≥ 10mm misclassified as < 10mm | 227/1,757 (12.9) |
| < 10mm misclassified as ≥ 10mm | 0/1,993 (0.0) |
| Total | 271/3,750 (7.2) |

**Supplementary Table 3.** Baseline characteristics of simulated polyps

| Polyp | Size, mm | Size category, mm | Morphology | Location | Shape | Height, mm |
| --- | --- | --- | --- | --- | --- | --- |
| S1 | 8.34 | 5–10 | 0-IIa | proximal colon | regular | 2.5 |
| S2 | 7.59 | 5–10 | 0-Ⅰs | proximal colon | regular | 4.5 |
| S3 | 7.15 | 5–10 | 0-Ⅰsp | proximal colon | regular | 7.0 |
| S4 | 7.75 | 5–10 | 0-Ⅰp | proximal colon | regular | 11.0 |
| S5 | 10.77 | ≥ 10 | 0-Ⅰsp | distal colon | regular | 9.5 |
| S6 | 9.08 | 5–10 | 0-Ⅰp | distal colon | irregular | 13.0 |
| S7 | 4.74 | < 5 | 0-Ⅰs | proximal colon | regular | 3.0 |
| S8 | 4.77 | < 5 | 0-Ⅰsp | distal colon | regular | 4.5 |
| S9 | 4.97 | < 5 | 0-Ⅰp | distal colon | regular | 8.5 |
| S10 | 10.70 | ≥ 10 | 0-Ⅰp | proximal colon | regular | 13.0 |

**Supplementary Table 4.** Baseline analysis of simulated polyps.

| Characteristic | *n* =10 |
| --- | --- |
| Size category, *n* (%) |  |
| < 5 mm | 3 (30) |
| 5–10 mm | 5 (50) |
| > 10 mm | 2 (20) |
| Morphology, *n* (%) |  |
| 0-Ip | 4 (40) |
| 0-Is | 2 (20) |
| 0-Ⅰsp | 3 (30) |
| 0-IIa | 1 (10) |
| Location, *n* (%) |  |
| Proximal colon | 6 (60) |
| Distal colon | 4 (40) |
| Shape, *n* (%) |  |
| Regular | 9 (90) |
| Irregular | 1 (10) |

**Supplementary Table 5.** Results of CAM system for real-time depth distance measurement.

| Field of view position | Depth distance | The 1st measurement | | | | | | The 2nd measurement | | | | | |
| --- | --- | --- | --- | --- | --- | --- | --- | --- | --- | --- | --- | --- | --- |
|  |  | Depth distance measurement, mean (IQR), mm | | $\text{ME}_{\text{Depth}}$, mm, mean (IQR) | $\text{PE}_{\text{Depth}}$, %, mean (IQR) | *P* value | CCC (95%CI) | Depth distance measurement, mm, mean (IQR) | | $\text{ME}_{\text{Depth}}$, mm, mean (IQR) | $\text{PE}_{\text{Depth}}$, %, mean (IQR) | *P* value | CCC (95%CI) |
|  |  | Graduated biopsy forceps | CAM system |  |  |  |  | Graduated biopsy forceps | CAM system |  |  |  |  |
| Center | Near | 12.3 (11.5–12.5) | 12.3 (10.1–14.2) | 2.2 (1.4–2.8) | 17.8 (10.6–23.4) | 0.26 | 0.12 (-0.63 to 0.87) | 12.2 (11.9–12.5) | 13.1 (11.1–14.4) | 1.8 (0.8–2.3) | 15.1 (6.0–18.5) | 0.03 | 0.01 (-0.75 to 0.76) |
|  | Medium | 24.9 (20.0–29.0) | 27.5 (22.8–32.2) | 3.0 (1.0–4.6) | 13.0 (3.2–22.6) | Reference | 0.71 (0.17–0.99) | 25.6 (23.3–28.5) | 25.4 (23.0–27.7) | 1.3 (0.6–1.8) | 5.3 (2.5–7.6) | Reference | 0.80 (0.35–0.99) |
|  | Far | 41.2 (39.1–42.6) | 41.3 (37.9–45.5) | 3.7 (2.4–5.1) | 8.9 (5.8–11.9) | 0.27 | 0.23 (-0.50 to 0.97) | 39.8 (37.3–42.5) | 37.4 (33.6–43.0) | 4.8 (2.3–6.6) | 11.8 (6.3–15.8) | 0.006 | 0.10 (-0.65 to 0.85) |
| Total | | 26.1 (12.0–40.3) | 27.0 (13.8–38.0) | 3.0 (1.7–4.2) | 13.2 (7.2–17.8) | 0.94 | 0.96 (0.85–0.99) | 25.9 (12.5–37.8) | 25.3 (13.9–33.8) | 2.6 (0.9–3.9) | 10.7 (3.5–14.7) | 0.049 | 0.95 (0.83 –0.99) |

IQR, interquartile range; CAM, computer-aided measuring; ${\text{M}\text{E}}_{\text{Depth}}$, measurement error of depth distance measurement; $\text{PE}_{\text{Depth}}$, percentage error of depth distance measurement; CCC, Lin’s concordance correlation coefficient between the depth distance measurement of CAM system and graduated biopsy forceps; CI, confidence interval.

**Supplementary Table 6.** Results of CAM system for real-time depth distance measurement in combining the 1st and 2nd measurements.

| Field of view position | Depth distance | Combining the 1st and 2nd measurements | | | | | |
| --- | --- | --- | --- | --- | --- | --- | --- |
|  |  | Depth distance measurement, mm, mean (IQR) | | $\text{ME}_{\text{Depth}}$, mm, mean (IQR) | $\text{PE}_{\text{Depth}}$, %,  mean (IQR) | *P* value | CCC (95%CI) |
|  |  | Graduated biopsy forceps | CAM system |  |  |  |  |
| Center | Near | 12.2 (11.5–12.5) | 12.7 (10.8–14.0) | 2.0 (1.0–2.7) | 16.5 (8.4–22.6) | 0.01 | 0.06 (-0.42 to 0.54) |
|  | Medium | 25.3 (22.5–28.5) | 26.5 (23.2–28.4) | 2.2 (0.7–3.5) | 9.1 (2.7–12.6) | Reference | 0.71 (0.38–0.99) |
|  | Far | 40.5 (38.1–42.5) | 39.3 (36.1–43.1) | 4.2 (2.5–5.8) | 10.4 (6.2–14.0) | 0.18 | 0.23 (-0.23 to 0.70) |
| Total | | 26.0 (12.5–38.4) | 26.2 (13.9–36.4) | 2.8 (1.1–4.1) | 12.0 (5.5–15.7) | 0.12 | 0.96 (0.88–0.99) |

IQR, interquartile range; CAM, computer-aided measuring; ${\text{M}\text{E}}_{\text{Depth}}$, measurement error of depth distance measurement; $\text{PE}_{\text{Depth}}$, percentage error of depth distance measurement; CCC, Lin’s concordance correlation coefficient between the depth distance measurement of CAM system and graduated biopsy forceps; CI, confidence interval.

**Supplemental Table 7.** Polyp size misclassification in different depth distances or field of view positions in the high-simulation colon/polyp model.

| The location of polyp size misclassifications | The 1st measurement  (*n* = 54) | The 2nd measurement  (*n* = 24) |
| --- | --- | --- |
| Medium-depth distance and within the central-FOV position | 0 (0.0) | 0 (0.0) |
| Depth distances |  |  |
| Near | 21 (38.9) | 10 (41.7) |
| Medium | 8 (14.8) | 4 (16.7) |
| Far | 25 (46.3) | 10 (41.7) |
| FOV position |  |  |
| Center | 2 (3.7) | 0 (0.0) |
| Top middle | 5 (9.3) | 2 (8.3) |
| Middle left | 7 (13.0) | 2 (8.3) |
| Middle right | 7 (13.0) | 3 (12.5) |
| Bottom middle | 6 (11.1) | 3 (12.5) |
| Top left | 6 (11.1) | 2 (8.3) |
| Top right | 7 (13.0) | 4 (16.7) |
| Bottom left | 6 (11.1) | 4 (16.7) |
| Bottom right | 8 (14.8) | 4 (16.7) |

FOV, field of view.

**Supplementary Table 8.** Average results of real-time polyp size measurement by near, medium, and far depth distances.

| Depth distance | Ground truth, mean (IQR), mm | The 1st measurement | | | | | The 2nd measurement | | | | | ICC (95%CI) |
| --- | --- | --- | --- | --- | --- | --- | --- | --- | --- | --- | --- | --- |
|  |  | CAM system  measurement, mm, mean (IQR) | $\text{ME}_{\text{Size}}$, mm, mean (IQR) | $\text{PE}_{\text{Size}}$, %, mean (IQR) | *P* value | CCC (95%CI) | CAM system  measurement, mm, mean (IQR) | $\text{ME}_{\text{Size}}$, mm, mean (IQR) | $\text{PE}_{\text{Size}}$, %, mean (IQR) | *P* value | CCC (95%CI) |  |
| Near_avg._ | 7.6 (4.9–9.5) | 7.5 (6.2–8.6) | 0.9 (0.8–1.3) | 11.9 (2.2–16.3) | 0.11 | 0.88 (0.52–0.99) | 7.8 (5.3–10.1) | 0.6 (0.3–0.8) | 8.6 (5.0–13.3) | 0.37 | 0.96 (0.75–0.99) | 0.94 (0.77–0.98) |
| Medium_avg._ |  | 7.7 (5.3–9.5) | 0.4 (0.2–0.7) | 5.5 (2.1–7.9) | Reference | 0.98 (0.85–0.99) | 7.8 (5.1–10.1) | 0.5 (0.2–1.0) | 6.8 (2.6–13.0) | Reference | 0.96 (0.75–0.99) | 0.99 (0.95–1.00) |
| Far_avg._ |  | 7.6 (5.7–9.5) | 0.8 (0.4–1.1) | 10.4 (5.4–16.9) | 0.04 | 0.93 (0.65–0.99) | 7.8 (5.6–10.0) | 0.6 (0.3–0.9) | 8.8 (3.2–16.8) | 0.41 | 0.96 (0.75–0.99) | 0.98 (0.91–0.99) |

avg., average; IQR, interquartile range; CAM, computer-aided measuring; $\text{ME}_{\text{Size}}$, measurement error of polyp size measurement; $\text{PE}_{\text{Size}}$, percentage error of polyp size measurement; CCC, Lin’s concordance correlation coefficient between the polyp size measurement of CAM system and ground truth; ICC, intraclass correlation coefficient between the polyp size measurement of the 1st and 2nd measurements; CI, confidence interval.

**Supplementary Table 9.** Average results of real-time polyp size measurement by 9 field of view positions.

| Field of view position | Ground truth, mm, mean (IQR) | The 1st time | | | | | The 2nd time | | | | | ICC (95% CI) |
| --- | --- | --- | --- | --- | --- | --- | --- | --- | --- | --- | --- | --- |
|  |  | CAM system  measurement, mm, mean (IQR) | $\text{ME}_{\text{Size}}$, mm, mean (IQR) | $\text{PE}_{\text{Size}}$, %, mean (IQR) | *P* value | CCC (95% CI) | CAM system  measurement, mm, mean (IQR) | $\text{ME}_{\text{Size}}$, mm, mean (IQR) | $\text{PE}_{\text{Size}}$, %, mean (IQR) | *P* value | CCC (95% CI) |  |
| Center_avg_ | 7.6 (4.9–9.5) | 7.5 (4.9–9.2) | 0.5 (0.1–0.8) | 5.8 (2.4–9.6) | Reference | 0.97 (0.80–0.99) | 7.6 (4.7–10.0) | 0.4 (0.2–0.4) | 5.3 (3.1–6.9) | Reference | 0.98 (0.81–0.99) | 0.99 (0.95–1.00) |
| Top middle_avg._ |  | 7.7 (5.9–9.2) | 0.6 (0.3–0.9) | 8.5 (2.9–11.8) | 0.24 | 0.97 (0.78–0.99) | 7.9 (5.3–10.1) | 0.5 (0.2–0.7) | 6.9 (2.8–11.3) | 0.19 | 0.96 (0.77–0.99) | 0.97 (0.88–0.99) |
| Middle left_avg._ |  | 7.5 (5.8–8.8) | 0.7 (0.3–1.1) | 9.8 (3.4–18.3) | 0.03 | 0.89 (0.54–0.99) | 7.8 (5.0–10.1) | 0.4 (0.2–0.5) | 5.5 (2.9–6.8) | 0.85 | 0.97 (0.79–0.99) | 0.95 (0.82–0.99) |
| Middle right_avg._ |  | 7.8 (6.5–8.9) | 0.8 (0.3–1.1) | 12.5 (4.2–16.8) | 0.09 | 0.93 (0.66–0.99) | 7.6 (5.6–10.1) | 0.8 (0.2–1.3) | 10.3 (3.0–18.4) | 0.054 | 0.91 (0.58–0.99) | 0.94 (0.76–0.99) |
| Bottom middle_avg._ |  | 7.5 (5.8–8.8) | 0.7 (0.3–1.1) | 9.2 (1.7–17.4) | 0.17 | 0.92 (0.63–0.99) | 8.0 (5.4–10.1) | 0.5 (0.3–0.7) | 7.5 (2.8–11.3) | 0.20 | 0.96 (0.76–0.99) | 0.96 (0.65–0.99) |
| Top left_avg._ |  | 7.4 (5.5–8.7) | 0.7 (0.1–1.1) | 8.8 (2.3–16.0) | 0.20 | 0.91 (0.59–0.99) | 7.8 (5.5–10.1) | 0.5 (0.1–0.7) | 7.0 (2.1–14.7) | 0.36 | 0.96 (0.76–0.99) | 0.95 (0.80–0.99) |
| Top right_avg._ |  | 7.7 (6.0–8.8) | 0.7 (0.2–1.1) | 9.9 (2.1–15.0) | 0.18 | 0.94 (0.70–0.99) | 7.9 (5.3–9.9) | 0.7 (0.3–1.2) | 9.3 (4.1–14.6) | 0.06 | 0.94 (0.68–0.99) | 0.96 (0.83–0.99) |
| Bottom left_avg._ |  | 7.5 (5.5–9.2) | 0.6 (0.2–0.9) | 7.8 (3.0–14.1) | 0.29 | 0.96 (0.73–0.99) | 8.2 (6.6–10.1) | 0.8 (0.3–1.1) | 12.8 (2.9–18.4) | 0.15 | 0.89 (0.55–0.99) | 0.92 (0.59–0.98) |
| Bottom right_avg._ |  | 7.9 (6.6–9.9) | 0.8 (0.2–1.1) | 12.2 (2.8–18.2) | 0.10 | 0.89 (0.55–0.99) | 7.6 (5.0–10.1) | 0.6 (0.2–1.1) | 8.3 (3.3–13.1) | 0.04 | 0.95 (0.71–0.99) | 0.95 (0.82–0.99) |

avg., average; IQR, interquartile range; CAM, computer-aided measuring; $\text{ME}_{\text{Size}}$, measurement error of polyp size measurement; $\text{PE}_{\text{Size}}$, percentage error of polyp size measurement; CCC, Lin’s concordance correlation coefficient between the polyp size measurement of the CAM system and ground truth; ICC, intraclass correlation coefficient between the polyp size measurement of the 1st and 2nd measurements; CI, confidence interval.

**Supplementary Table 10.** Results of real-time polyp size measurement by near-medium-far depth distances and within 9 field of view positions.

| Field of view position | Depth distance | Ground truth, mm, mean (IQR) | The 1st time | | | | | The 2nd time | | | | | ICC (95% CI) |
| --- | --- | --- | --- | --- | --- | --- | --- | --- | --- | --- | --- | --- | --- |
|  |  |  | CAM system  measurement, mm, mean (IQR) | $\text{ME}_{\text{Size}}$, mm, mean (IQR) | $\text{PE}_{\text{Size}}$, mean, % (IQR) | *P* value | CCC (95% CI) | CAM system  measurement, mm, mean (IQR) | $\text{ME}_{\text{Size}}$, mm, mean (IQR) | $\text{PE}_{\text{Size}}$, mean (IQR), % | *P* value | CCC (95% CI) |  |
| Center | Near | 7.6 (4.9–9.5) | 7.2 (4.8–8.1) | 0.5 (0.1–0.9) | 6.1 (1.6–9.9) | 0.34 | 0.93 (0.65–0.99) | 7.6 (4.8–10.1) | 0.4 (0.1–0.6) | 4.8 (1.8–6.1) | 0.49 | 0.96 (0.76–0.99) | 0.90 (0.66–0.97) |
|  | Medium |  | 7.7 (4.8–10.0) | 0.3 (0.1–0.3) | 4.1 (2.6–4.5) | Reference | 0.98 (0.82–0.99) | 7.7 (4.8–9.7) | 0.3 (0.1–0.3) | 3.3 (1.8–3.5) | Reference | 0.99 (0.87–0.99) | 0.999 (0.994–1.000) |
|  | Far |  | 7.6 (5.4–9.1) | 0.6 (0.2–0.9) | 7.1 (2.8–15.4) | 0.04 | 0.92 (0.64–0.99) | 7.4 (4.6–10.1) | 0.5 (0.3–0.8) | 7.8 (3.3–12.1) | 0.04 | 0.96 (0.75–0.99) | 0.93 (0.75–0.98) |
| Top  middle | Near |  | 7.4 (5.2–8.8) | 1.0 (0.2–2.0) | 13.9 (2.8–21.2) | 0.14 | 0.76 (0.28–0.99) | 8.0 (6.3–10.0) | 0.5 (0.1–0.6) | 7.7 (1.2–6.7) | 0.41 | 0.93 (0.65–0.99) | 0.66 (0.12–0.90) |
|  | Medium |  | 7.6 (5.5–9.4) | 0.3 (0.1–0.4) | 4.6 (1.4–4.8) | 0.81 | 0.98 (0.83–0.99) | 7.7 (4.8–10.1) | 0.3 (0.04–0.3) | 3.6 (0.8–3.4) | 0.24 | 0.98 (0.81–0.99) | 0.95 (0.80–0.99) |
|  | Far |  | 7.9 (5.8–10.1) | 0.5 (0.1–0.9) | 7.1 (2.6–16.9) | 0.11 | 0.96 (0.74–0.99) | 7.9 (5.5–10.1) | 0.7 (0.2–1.1) | 9.5 (2.9–18.0) | 0.08 | 0.91 (0.61–0.99) | 0.94 (0.77–0.98) |
| Middle left | Near |  | 7.8 (6.5–9.1) | 0.9 (0.1–1.8) | 13.3 (1.1–20.1) | 0.15 | 0.74 (0.24–0.99) | 7.7 (4.9–10.1) | 0.3 (0.1–0.3) | 3.7 (1.2–3.6) | 0.15 | 0.98 (0.81–0.99) | 0.78 (0.31–0.94) |
|  | Medium |  | 7.5 (5.6–9.1) | 0.7 (0.1–1.3) | 8.5 (1.2–17.6) | 0.48 | 0.90 (0.57–0.99) | 7.9 (4.8–10.1) | 0.5 (0.1–0.6) | 6.8 (2.1–7.0) | 0.48 | 0.93 (0.66–0.99) | 0.84 (0.50–0.96) |
|  | Far |  | 7.3 (4.8–9.1) | 0.7 (0.1–1.5) | 7.6 (2.0–16.6) | 0.17 | 0.90 (0.58–0.99) | 7.7 (5.4–10.0) | 0.4 (0.2–0.8) | 6.0 (2.6–11.9) | 0.17 | 0.96 (0.77–0.99) | 0.94 (0.75–0.98) |
| Middle right | Near |  | 7.8 (7.1–8.8) | 1.0 (0.2–1.7) | 15.3 (2.1–21.0) | 0.09 | 0.75 (0.24–0.99) | 7.5 (5.0–10.1) | 1.0 (0.1–2.1) | 13.5 (2.0–26.9) | 0.06 | 0.80 (0.36–0.99) | 0.54 (-0.12 to 0.86) |
|  | Medium |  | 7.6 (5.5–9.4) | 0.3 (0.1–0.4) | 4.6 (1.2–5.9) | 0.80 | 0.98 (0.83–0.99) | 7.7 (5.5–10.1) | 0.9 (0.1–1.6) | 10.9 (2.1–20.1) | 0.13 | 0.82 (0.40–0.99) | 0.82 (0.41–0.95) |
|  | Far |  | 7.9 (6.3–10.1) | 1.1 (0.2–2.1) | 17.6 (2.6–35.3) | 0.07 | 0.77 (0.30–0.99) | 7.7 (5.5–10.1) | 0.5 (0.1–0.9) | 6.5 (2.0–12.6) | 0.25 | 0.96 (0.74–0.99) | 0.86 (0.55–0.96) |
| Bottom  middle | Near |  | 6.7 (4.8–9.1) | 1.2 (0.1–2.1) | 15.4 (2.0–25.6) | 0.06 | 0.71 (0.18–0.99) | 8.2 (4.9–10.1) | 0.7 (0.1–1.5) | 9.6 (2.0–18.9) | 0.09 | 0.90 (0.57–0.99) | 0.69 (0.02–0.92) |
|  | Medium |  | 7.8 (5.4–10.1) | 0.4 (0.1–0.4) | 5.1 (1.5–7.0) | 0.53 | 0.97 (0.78–0.99) | 7.7 (4.9–10.1) | 0.3 (0.05–0.3) | 3.4 (0.6–3.2) | 0.91 | 0.98 (0.81–0.99) | 0.99 (0.97–1.00) |
|  | Far |  | 7.6 (5.5–9.1) | 0.5 (0.1–1.1) | 7.0 (1.4–17.6) | 0.22 | 0.92 (0.62–0.99) | 8.0 (6.6–10.1) | 0.6 (0.1–0.9) | 9.3 (2.1–15.4) | 0.11 | 0.92 (0.62–0.99) | 0.87 (0.58–0.97) |
| Top  left | Near |  | 7.4 (6.4–8.2) | 0.8 (0.1–1.2) | 10.1 (1.2–14.5) | 0.33 | 0.81 (0.38–0.99) | 8.0 (6.4–10.0) | 0.5 (0.1–0.6) | 7.6 (1.2–6.7) | 0.34 | 0.93 (0.64–0.99) | 0.81 (0.44–0.95) |
|  | Medium |  | 7.5 (4.8–9.1) | 0.5 (0.1–0.6) | 5.4 (1.8–7.0) | 0.41 | 0.93 (0.66–0.99) | 7.7 (4.8–10.1) | 0.3 (0.1–0.3) | 3.9 (1.8–3.5) | 0.32 | 0.98 (0.81–0.99) | 0.96 (0.85–0.99) |
|  | Far |  | 7.3 (5.5–8.8) | 0.8 (0.1–1.6) | 10.1 (1.8–17.9) | 0.04 | 0.83 (0.40–0.99) | 7.8 (5.4–10.1) | 0.6 (0.1–1.5) | 9.3 (2.1–18.2) | 0.11 | 0.92 (0.61–0.99) | 0.80 (0.41–0.95) |
| Top right | Near |  | 7.6 (6.8–8.8) | 0.9 (0.2–1.7) | 13.5 (2.7–19.1) | 0.13 | 0.78 (0.31–0.99) | 7.9 (6.4–10.1) | 0.5 (0.05–0.6) | 8.0 (0.6–8.1) | 0.30 | 0.92 (0.64–0.99) | 0.66 (0.09–0.90) |
|  | Medium |  | 7.8 (5.7–9.4) | 0.3 (0.1–0.4) | 5.2 (1.6–6.3) | 0.67 | 0.98 (0.83–0.99) | 7.8 (5.5–10.1) | 0.9 (0.1–1.6) | 11.4 (2.1–20.4) | 0.08 | 0.82 (0.38–0.99) | 0.83 (0.43–0.95) |
|  | Far |  | 7.8 (6.5–9.1) | 0.7 (0.1–1.6) | 10.9 (1.8–18.2) | 0.13 | 0.86 (0.48–0.99) | 7.8 (5.5–10.1) | 0.6 (0.2–0.9) | 8.4 (3.0–13.1) | 0.15 | 0.93 (0.65–0.99) | 0.83 (0.44–0.95) |
| Bottom left | Near |  | 7.5 (4.8–9.1) | 0.5 (0.1–0.6) | 5.3 (1.4–7.0) | 0.47 | 0.93 (0.66–0.99) | 8.1 (6.4–10.1) | 0.7 (0.1–1.2) | 10.5 (1.8–14.5) | 0.24 | 0.87 (0.51–0.99) | 0.85 (0.51–0.96) |
|  | Medium |  | 7.7 (5.5–10.1) | 0.4 (0.1–0.9) | 6.1 (1.1–12.6) | 0.04 | 0.96 (0.74–0.99) | 8.4 (5.7–11.0) | 0.8 (0.2–1.6) | 11.6 (2.9–19.9) | 0.03 | 0.89 (0.55–0.99) | 0.89 (0.58–0.97) |
|  | Far |  | 7.4 (6.6–8.8) | 0.9 (0.2–1.9) | 12.2 (2.8–18.8) | 0.11 | 0.78 (0.31–0.99) | 8.2 (6.9–10.1) | 0.9 (0.2–1.6) | 16.3 (2.6–23.8) | 0.07 | 0.75 (0.26–0.99) | 0.49 (-0.08 to 0.84) |
| Bottom right | Near |  | 7.8 (6.6–9.0) | 0.9 (0.2–1.6) | 13.9 (1.9–19.1) | 0.11 | 0.78 (0.30–0.99) | 7.4 (4.8–10.1) | 1.0 (0.1–2.2) | 12.2 (2.5–25.3) | 0.07 | 0.82 (0.39–0.99) | 0.72 (0.20–0.92) |
|  | Medium |  | 7.8 (5.5–10.1) | 0.4 (0.1–0.5) | 5.7 (1.4–7.5) | 0.40 | 0.96 (0.76–0.99) | 7.7 (5.4–10.1) | 0.5 (0.1–0.8) | 6.1 (2.1–12.6) | 0.11 | 0.96 (0.74–0.99) | 0.98 (0.92–0.99) |
|  | Far |  | 8.1 (7.0–9.1) | 1.0 (0.2–2.0) | 17.0 (2.1–26.4) | 0.07 | 0.71 (0.18–0.99) | 7.8 (5.4–10.1) | 0.4 (0.1–0.6) | 6.4 (2.1–11.5) | 0.07 | 0.96 (0.76–0.99) | 0.76 (0.32–0.94) |
| Average | |  | 7.6 (5.6–9.2) | 0.7 (0.4–1.0) | 9.4 (5.2–14.8) | 0.01 | 0.96 (0.74–0.99) | 7.8 (5.3–10.0) | 0.6 (0.3–0.7) | 8.1 (4.4–10.4) | 0.002 | 0.97 (0.77–0.99) | 0.98 (0.92–1.00) |
| Total | | 7.6 (5.0–9.1) | 7.6 (5.7–8.8) | 0.7 (0.2–1.0) | 9.4 (1.9–17.5) | 0.37 | 0.87 (0.82–0.93) | 7.8 (5.7–9.8) | 0.6 (0.2–0.9) | 8.3 (2.1–13.5) | 0.22 | 0.91 (0.86–0.96) | 0.82 (0.77–0.85) |

IQR, interquartile range; CAM, computer-aided measuring; $\text{ME}_{\text{Size}}$, measurement error of polyp size measurement; $\text{PE}_{\text{Size}}$, percentage error of polyp size measurement; CCC, Lin’s concordance correlation coefficient between the polyp size measurement of CAM system and ground truth; ICC, intraclass correlation coefficient between the polyp size measurement of the 1st and 2nd measurements; CI, confidence interval.

**Supplemental Table 11.** Polyp size misclassification according to the cut-off of 5 and 10 mm in the high-simulation colonoscopy model.

| Polyp size misclassifications | The 1st measurement, *n* (%) | The 2nd measurement, *n* (%) |
| --- | --- | --- |
| ≥ 5mm misclassified as < 5mm | 3/189 (1.6) | 1/189 (0.5) |
| < 5mm misclassified as ≥ 5mm | 31/81 (38.3) | 22/81 (27.2) |
| ≥ 10mm misclassified as < 10mm | 20/54 (37.0) | 0/54 (0.0) |
| < 10mm misclassified as ≥ 10mm | 0/216 (0.0) | 1/216 (0.5) |
| Total | 54/270 (20.0) | 24/270 (8.9) |

**Supplementary Table 12.** Dataset summary of polyp detection model.

| Dataset | Category | Images | Source | Findings |
| --- | --- | --- | --- | --- |
| Kvasir-SEG | Public | 1,000 | WL | Polyp |
| CVC-ClinicDB | Public | 612 | WL | Polyp |
| CVC-ColonDB | Public | 380 | WL | Polyp |
| CVC-300 | Public | 60 | WL | Polyp |
| ETI-LaribPolypDB | Public | 196 | WL | Polyp |
| EndoCV2022 | Public | 3,292 | WL NBI | Polyp |
| EDD2020 | Public | 386 | WL NBI | Polyp |
| TSGH | Private | 3,317 | WL NBI | Polyp |
| SCMH | Private | 2,944 | WL NBI | Polyp |
| PICCOLO (Testing) | Public | 3,433 | WL NBI | Adenoma hyperplastic |

WL, white light; NBI, narrow band imaging.

**Supplementary Table 13.** The analysis of polyp detection on the PICCOLO dataset.

| Specificity | FP counts | Sensitivity | FN counts |
| --- | --- | --- | --- |
| 94.63% | 54 | 92.92% | 243 |

FP, false positive; FN, false negative.

**Supplementary Table 14.** Performance metrics of polyp detection on the total image test dataset, for different subsets according to the histology, morphology, size of the polyp.

| Variable | Recall | Precision | F1-Score |
| --- | --- | --- | --- |
| Total | 0.929 | 0.969 | 0.949 |
| Histology |  | | |
| Adenoma | 0.958 | 0.954 | 0.956 |
| Hyperplasia | 0.807 | 0.843 | 0.825 |
| Adenocarcinoma | 0.966 | 0.813 | 0.883 |
| Morphology |  | | |
| Protruded | 0.977 | 0.926 | 0.951 |
| Flat | 0.881 | 0.934 | 0.906 |
| Size |  | | |
| ≥ 5mm | 0.927 | 0.956 | 0.941 |
| < 5mm | 0.895 | 0.809 | 0.850 |
